# Four fundamental dimensions underlie the perception of human actions

**DOI:** 10.3758/s13414-023-02709-1

**Published:** 2023-05-15

**Authors:** Laura C. Vinton, Catherine Preston, Stephan de la Rosa, Gabriel Mackie, Steven P. Tipper, Nick E. Barraclough

**Affiliations:** 1 Department of Psychology, University of York, Heslington, York, YO10 5DD UK; 2 Department of Social Sciences, IU University of Applied Sciences, Juri-Gagarin-Ring 152, 99084 Erfurt, Germany

**Keywords:** Action, Perception, Model, Conceptual space, Representation

## Abstract

We evaluate the actions of other individuals based upon a variety of movements that reveal critical information to guide decision making and behavioural responses. These signals convey a range of information about the actor, including their goals, intentions and internal mental states. Although progress has been made to identify cortical regions involved in action processing, the organising principles underlying our representation of actions still remains unclear. In this paper we investigated the conceptual space that underlies action perception by assessing which qualities are fundamental to the perception of human actions. We recorded 240 different actions using motion-capture and used these data to animate a volumetric avatar that performed the different actions. 230 participants then viewed these actions and rated the extent to which each action demonstrated 23 different action characteristics (e.g., avoiding-approaching, pulling-pushing, weak-powerful). We analysed these data using Exploratory Factor Analysis to examine the latent factors underlying visual action perception. The best fitting model was a four-dimensional model with oblique rotation. We named the factors: friendly-unfriendly, formidable-feeble, planned-unplanned, and abduction-adduction. The first two factors of friendliness and formidableness explained approximately 22% of the variance each, compared to planned and abduction, which explained approximately 7–8% of the variance each; as such we interpret this representation of action space as having 2 + 2 dimensions. A closer examination of the first two factors suggests a similarity to the principal factors underlying our evaluation of facial traits and emotions, whilst the last two factors of planning and abduction appear unique to actions.

**Supplementary Information:**

The online version contains supplementary material available at 10.3758/s13414-023-02709-1.

## Introduction

The organising principal underlying our mental representations is that features of our external world are represented in different internal workspaces or ‘conceptual spaces’ (Allen, 1984; Gärdenfors, 2004b). These spaces capture the similarities and differences between items of a domain and enable further classification, naming and responses to the information. Uncovering the organisation of psychological, and underlying neural, representations of our external world has been fundamental to progress in psychology and neuroscience over recent decades (Gärdenfors, 2004b; Shepard, 1987).

Evidence suggests that items from different domains and across several different modalities are represented within their own conceptual spaces, such as colour (Bonnardel et al., 2016), face identity (Catz et al., 2009; Nishimura et al., 2009), face traits (Oosterhof & Todorov, 2008; Sutherland et al., 2013), sound effects (Scavone et al., 2001), odours (Bao et al., 2019), tactile textures (Hollins et al., 2000) and the taste of wine, (Ballester et al., 2005; Ballester et al., 2008). The structure of each conceptual space is specific to the domain, with items in the domain that are perceived to be more similar being positioned closer to each other within the conceptual space, whilst dissimilar items are located further apart. For example, colours that are more similar, such as blue and turquoise, are positioned closer within colour space than less similar colours, such as blue and orange (Bonnardel et al., 2016). Although items in a domain can be evaluated on a wide range of different characteristics, not all characteristics are fundamental to the representation of the domain. Instead patterns of similarity among items or clusters of similar items determine the dimensions that structure the conceptual space (Gärdenfors, 2004a). These dimensions are the fundamental factors that define the structure of the conceptual space, and the meaningful information on which we make decisions about the items. For example, we can evaluate faces on a range of different characteristics including trustworthiness, dominance, youthful attractiveness, sexual dimorphism, intelligence and confidence (Oosterhof & Todorov, 2008; Sutherland et al., 2013). However, it appears we evaluate face traits principally on the underlying fundamental factors of trustworthiness and dominance (Oosterhof & Todorov, 2008). Similar results identifying these two factors have been observed by Sutherland et al. (2013); however, their use of a more varied range of naturalistic faces suggested an additional third factor of youthful attractiveness was also important. Uncovering these fundamental dimensions of face trait space has led to further theoretical advances, including understanding preconscious face perception (Stewart et al., 2012), and dissociating the role of different neural structures underlying face processing (Getov et al., 2015).

Gärdenfors and Warglien (2012) proposed that human actions might also be represented within a conceptual space, as an ‘action space’. They suggested that action space would represent principally the movement, as such the kinematics of the body, the forces exerted at each body and limb joint, and the spatio-temporal properties. However, we evaluate actions on a range of different characteristics; for example, the action of hugging another individual can be understood in terms of the action kinematics (lifting both arms in front of the body followed by articulation at the elbows), in terms of the action goals (to grasp another individual close to the body), or in terms of the actor’s intentions (to console another individual). The ability to perceive and understand the actions of other individuals in these different ways is crucial to our memory of our social environment and guides us in how to respond optimally to other people (Becchio et al., 2010; Blake & Shiffrar, 2007; Knoblich & Sebanz, 2006; Macrae et al., 2008). These higher, more abstract levels of evaluation including action goals and actor intentions would not be accounted for within the Gärdenfors and Warglien (2012) action space proposal, which centred principally around body movements.

Instead, action space may additionally represent these higher levels of abstraction, as suggested by different theoretical models suggesting that actions are represented at multiple levels (e.g., Ciaramidaro et al., 2007; Hamilton & Grafton, 2006; Ondobaka & Bekkering, 2012; Van Overwalle & Baetens, 2009; Wurm & Lingnau, 2015). Most proposals suggest that we evaluate actions on their movement (kinematic or spatio-temporal properties), actions goals and actor intentions. However, they can disagree as to the number of levels on which actions can be understood, how many sub-levels underlie understanding of action goals and actor intentions, and which neural substrates underpin these different processes. For example, Hamilton and Grafton (2006) separate action goals into ‘immediate goals’ and ‘task goals’, Ciaramidaro et al. (2007) distinguish private and social goals and intentions, whilst Wurm and Lingnau (2015) refer to concrete, intermediate and abstract levels of action understanding. Furthermore, there is likely to be an interaction between how we understand these actions at these different levels given the interplay between the processing of the different types of action information (e.g., Gunns et al., 2002; Loucks & Pechey, 2016; Montepare et al., 1987; Paterson et al., 2001). Therefore, any comprehensive action space is likely to not just represent body movements, but also more abstract information about the purpose of actions and their motivation.

Various prior attempts have been made to assess action space; however, these have either not attempted to assess which fundamental dimensions underly action space, or not always taken into account the unique qualities of the action domain. Giese et al. (2008) have shown that actions can be represented within a low-dimensional perceptual space, and the structure of this action representation is closely related to the physical similarity of the movement of the joints of the actor. However, the limited number and type of actions tested (types of locomotion) couldn’t allow an assessment of the dimensionality of a more general action space. Some have taken a different approach, by assessing actions defined in the very widest sense (‘discrete, meaningful events caused by one or more human, living, or non-living entity’ (Thornton & Tamir, 2022). Their approach was to assess how we organise our understanding of verbs (as proxies for actions), and identified six separate dimensions underlying their representation: Abstraction, Creation, Tradition, Food, Animacy and Spiritualism. However, some of these verbs were either particularly abstract or could not be motorically executed by human beings, whilst other verbs described complex sequences of activities. It is not, therefore, immediately clear how this framework that was built on the evaluation of such a diverse set of verbs might relate to a conceptual space that represents solely the domain of discrete visible human actions (Wurm & Caramazza, 2019). Others have examined the organisation of the neural representation of images of actions by combining functional magnetic resonance imagining (fMRI) and behavioural methods (Tucciarelli et al., 2019). Here, they asked participants to arrange images of 28 actions within a two-dimensional space in five different ways according to: the semantic similarity of the actions, the body parts involved in the actions, the likely context in which the actions occur, the type of movement involved in the action, and the type of object involved in the actions. The pattern of activity within the action observation network (Decety & Grèzes, 1999) was best represented by the organisation of actions according to semantic similarity. Principal components analysis suggested that when participants organised the actions by semantic meaning, three main components could explain the majority of observed variance. Although this analysis could not identify appropriate labels for the three dimensions, it did suggest actions may be distinguished according to: the type of change induced by the action, the type of need fulfilled by the action, and the degree to which the action is directed towards another person. Whilst such a three-dimensional model of action organisation may best explain the data of Tucciarelli et al. (2019), the lack of movement present in their stimuli, the relatively small set of actions, and the constraints of the task where participants only organised actions based upon semantic similarity, may have limited this study’s ability to fully reveal other potential dimensions and the organising principles of an action space (Tucciarelli et al., 2019).

In this paper we aimed to determine the conceptual space that underlies action perception by assessing which action qualities are fundamental to the perception of actions executed by other human individuals. Our approach was to use a data-driven method similar to Sutherland et al. (2013), who determined face trait space from assessments of a diverse range of naturalistic faces. We wanted to assess how people perceive actions in as broad a range as possible from multiple levels of abstraction that can be used to evaluate actions, from the movement to the goals and intentions. Similar to Sutherland et al. (2013), we asked participants to evaluate 240 diverse dynamic whole-body actions on 23 different characteristics and then used an exploratory factor analysis to determine the latent factors underlying action perception. (Howard, 2016; Schmitt, 2011).

## Methods

### Stimuli

**Fig. 1:**
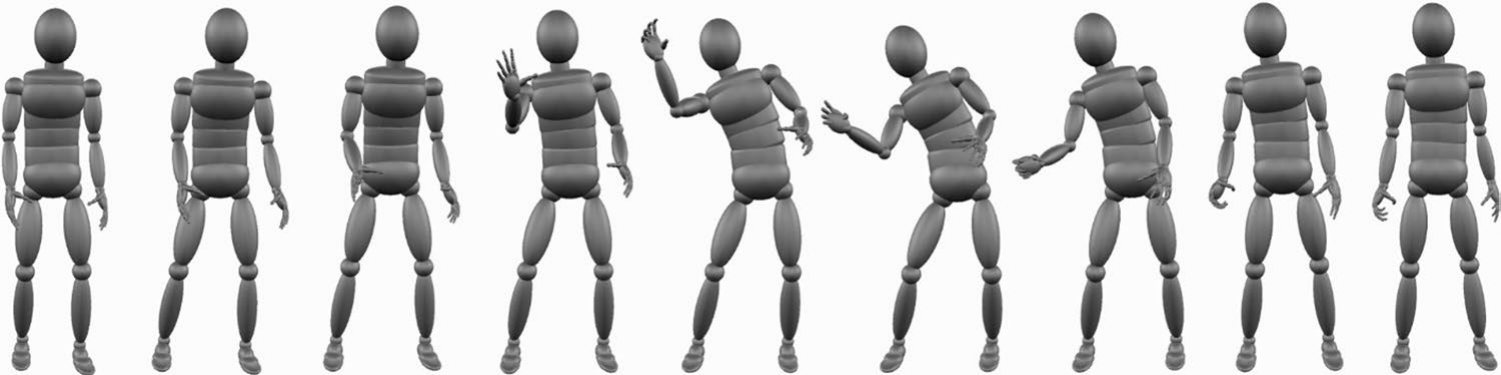
Illustration of frames (1, 13, 25, 37,49, 61, 73, 85, 97) from the catching action

Two hundred and forty different actions were chosen to represent a broad a range of action types as possible based upon the examination of 12 different databases of actions (see Online Supplementary Materials (OSM) for a full list), as well as additional deliberate and accidental actions opportunistically recorded during the recording process. Actions were recorded (at 60 frames per second (fps)) from four actors (two female) using a 32-channel motion capture suit (Noitom, Noitom International, Inc., FL. USA). Each of the 240 actions were performed by both a male and a female actor (480 actions in total, 120 actions per actor). In addition, actors were asked to perform three versions of each of their 120 actions in a fashion that the actor felt was most natural. Those actions that were transitive were executed whilst interacting with appropriate objects to ensure that they were performed in a natural fashion. However, the movements of the objects were not captured and objects were not present in the resulting actions used in the experiment (the actions appeared ‘pantomimed’) in order to isolate only perceptual information from the bodies and body movements of actors. In total we recorded 1,440 action exemplars (240 actions x 2 genders x 3 versions). Each of these actions was subjected to quality checks, pre-processing and cropping, so that the recording only featured the most typical presentation of the intended action (see OSM for details). In order to select the final 240 action stimuli, each of the 1,440 actions were presented on-screen by an androgynous volumetric avatar (see OSM) using Unity 3D (Unity, San Francisco, CA. USA). Three of the authors (LV, NB, GM) evaluated all of the actions on the basis of whether they were representative of the intended action (on a 1–9 Likert scale), and whether the motion capture recording was of good quality (yes/no). These data were first used to eliminate those action exemplars that were of poor quality. Two of these actions (crossing arms and touching abdomen (stomach-ache)) were rated as poor quality by all reviewers, and were therefore replaced by additional actions (failing to catch a ball and petting a dog). Second, the maximal average rating of each action type executed by a male and a female actor was used to select the best 480 actions that represented the intended action (240 male, 240 female actions). Finally, for actions where the male and female examples were rated the same, the examples were pseudo-randomly selected so that the final stimuli set of 240 action recordings contained equal numbers of actions performed by male and female actors, were closely balanced for actions from the different actors, and were closely balanced for social actions and whether the actions were transitive (see Table 1). The final 240 action stimuli selected were all seen as good quality recordings and showed high average representativeness (mean = 8.075; minimum = 6.333, maximum = 9). In order to ensure that our final actions used in the experiment were brief and showed only a single action, we cropped (in time) the actions to eliminate early standing and preparatory actor movements (e.g., approaching objects/people, picking up a knife before executing a cutting action) and later retreating movements of the actors. This resulted in the final 240 actions ranging in duration between 1.67 s and 3 s, 100–180 frames at 60 fps (mean duration = 2.5 s, SD = 0.46 s). Finally, to generate files to use in the online experiment, actions were then rendered on-screen using Unity 3D (Unity, San Francisco, CA. USA) with the avatar for each action positioned in the centre of the screen, with adjustments made to travelling actions to ensure they remained fully visible for the duration of the action. Actions were captured (Bailey & OBS Studio Contributors, 2020), and then edited using DaVinci Resolve16 (Blackmagic Design, Melbourne, Victoria, Australia) to generate 240 separate .mp4 files (each 1,280 x 1,080 pixels, 60 fps, H.264 codec with Network Optimisation to allow for faster streaming). Figure 1 shows an example action, whilst all actions used during the rating experiment can be found freely available online at the Open Science Framework (https://osf.io/4vew8/).

**Table 1:** Distribution of actions included in the final set of 240, across actors, sociality, and transitivity

Sex	Actor		Sociality		Transitivity	
	1st	2nd	Social	Non-social	Transitive	Non-transitive
Female	50	70	40	80	46	74
Male	53	67	35	85	47	73

### Action recognition

In order to determine how well our 240 actions (listed at https://osf.io/4vew8/) were recognised as reflecting the goals and intentions of the actors during the motion capture process, we asked naïve observers to take part in a separate online experiment, implemented via the Gorilla Experiment Builder (Anwyl-Irvine et al., 2019; Anwyl-Irvine et al., 2021). Participants (n = 30; 27 female, two male, one other/prefer not to say; mean age = 19.07 years, SD age = .74 years) were recruited through the internal University of York SONA system and compensated with course credit (n = 28) or recruited opportunistically (n = 2). A power analysis was not appropriate in this instance as we did not conduct any inferential statistics on the data; however, the central limit theorem states that a sample size of 30 would be appropriate for most distributions. All participants provided informed consent, and the experiment was approved by the ethics committee of the Department of Psychology, University of York, and was performed in accordance with the ethical standards laid down in the 1964 Declaration of Helsinki.

During the experiment participants were presented with each of the 240 action videos, and for each one they were required to “type as many names as you can think of that would apply to the action in the video”. During a trial, participants were presented with the action video on-screen that they could click on to make it play up to three times in total. To the side of the action video were six text boxes where they could enter up to six responses. Once they were happy with their response, they could press a button to continue to the next trial. Breaks were given every 60 actions to help maintain concentration throughout the duration of the task. The experiment took on average 60 min to complete.

In total the 30 participants assigned 14,454 names to the 240 actions; each participant therefore assigned on average 2.01 names to each action. Analysis of the data was performed in order to determine whether observers could recognise the purpose of actions carried out by the original actors, even when they were conveyed by a synthetic volumetric puppet without any social (other actors) or physical context (e.g., relevant objects pertinent to the execution of transitive actions). Initial categorisation of participant names was performed by one of the authors, who fully understood the action goals and actor intentions when they were executed by the original actors during the motion capture process. The names provided by participants for each action were compared against a full description of the actors’ original goals and intentions from the acting and motion-capture process. A score of 1 was assigned if a participant’s name was an ‘exact match’ to either the purpose of the action or the intention of the actor. If names provided merely a description of movements of body parts (e.g., ‘moving hands’) and did not indicate that the observer recognised the goal or intention of the actor, this was scored as 0. Names that were similar, but not an exact match, were flagged as ‘possible matches’ (274 in total). These names were then assessed against the full description of the actors’ original goals and intentions by another naïve individual. If they judged that the names were good matches, then these names were also scored as 1; if not these names were assessed by a third naïve individual. If this third individual judged that the names were good matches, then these names were also scored as 1; finally, all remaining names were scored as 0. This process was to ensure that all ‘possible matches’ were only scored as 1 if two individuals (author + 1 naïve individual) agreed (77/274 possible matches were consequently scored as matches following this process).

**Fig. 2:**
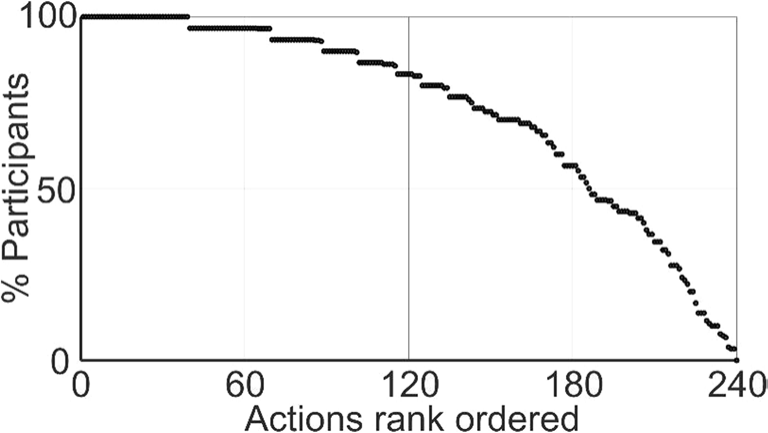
Recognition of action goals and intentions. Actions are rank ordered based upon how well they were recognised to form a recognisability spectrum along the x axis. Each dot represents a single action; the y axis indicates the percentage of participants that accurately recognised the actions

For each of the 240 actions, scores were counted across the 30 participants, calculated as a percentage of total possible scores and then rank ordered (illustrated in Fig. 2). At one end of this ‘recognisability spectrum’, 39 actions were recognised by all participants (30 matched names from 30 participants), whilst a further 30 actions were recognised by 29 of 30 participants. At the other end of this spectrum, one action (Laughing menacingly) was not recognised by anyone, whilst a few actions were only recognised by a small number of participants. These tended to be transitive actions involving the careful manipulation of specific objects (see OSM for a fuller exposition). Importantly, however, 77.5% of all actions were recognised by the majority of participants, whist the median action on the recognisability spectrum was recognised by 83% of participants. Importantly, these names were not used during the rating experiment on which the model of action space is based.

### Selection of action characteristics

In order to choose the action characteristics on which the actions were to be rated in the main experiment, we first aimed to establish as comprehensive a range of characteristics as possible that people use to evaluate actions. To do this, we first selected a broad range (in terms of actions, actors, contexts, viewpoints) of 500 different photorealistic videos of naturalistic actions available in a number of openly available online action databases (see OSM for list). These video dimensions varied in size and duration (widths 160–720 pixels x heights 120–576 pixels, durations 0.85–24 s). All videos were converted (using ffmpeg; https://ffmpeg.org/) to .mp4 format, audio information removed, and saved at a framerate of 25 fps.

Action characteristics were then determined in an unconstrained characteristic identification experiment as per Oosterhof and Todorov (2008). 106 participants (41 recruited via social media, 65 for course credit) completed the experiment remotely via an online experimental survey tool (Qualtrics 2018, Provo, UT, USA). No inferential statistical tests were planned for these data, and so a power analysis was not appropriate here. Instead, the number of participants was chosen based upon prior research as per Oosterhof and Todorov (2008). The experiment was approved by the ethics committee of the Department of Psychology, University of York and performed in accordance with the ethical standards laid down in the 1990 Declaration of Helsinki; all participants provided informed consent.

The 500 naturalistic action videos were arbitrarily separated into ten blocks of 50 different actions, and participants were allocated to one of the ten blocks of 50 actions. In total, each block of 50 actions was seen by between 10 and 13 participants. Initially, participants viewed a consent form before viewing the actions. Subsequently, on each trial a pseudo-randomly selected action video was presented on a white screen, above which was the text “Describe what comes to mind when you see this action”, and below the action was a text box in which participants were to indicate their responses. The question asked was deliberately broad as actions can be understood on multiple levels of abstraction (Hamilton & Grafton, 2006; Van Overwalle & Baetens, 2009), and we didn’t want to constrain participants’ responses to just one interpretation of the stimulus. Participants had as long as they liked to indicate as many words as they liked associated with the action. Once they were happy with their response, they clicked a button on the screen to proceed to the next trial.

In total 13,539 words were reported by the 106 participants, of which 2,303 were unique. Initially, two individuals (author NB and a research assistant) independently classified these words into broad characteristics, and as per Oosterhof and Todorov (2008), and then subsequently met to agree upon a final list of 31 characteristics (see Table S3, OSM). Of those 31 characteristics, 23 related to the actions themselves (accounting for 70.20% of participant’s descriptions), whilst eight other characteristics related to non-action specific aspects of the stimuli (e.g., person descriptors, locations, personality traits, abstract concepts and objects).

Action characteristics ranged from two ends of a spectrum, for example ’weak-powerful‘, ’disapproving-approving‘, and ’lowering-raising‘. The characteristics included descriptions of simple kinematic properties of the action (e.g., low speed-high speed, fluent-hesitant and uncontrolled-controlled), to more abstract goals and intentions of the action, (e.g., making-breaking, rejecting-desiring, and threatening-protecting). In order to determine which of the two extremes should lie at which end of a Likert scale, a further 14 participants were asked to indicate which was the most intuitive position on the Likert scale for the two extremes of each action quality. The results indicated that for all 23 action characteristics between 64.3% and 100% of participants agreed upon position, and this order was used in the subsequent rating experiment.

### Rating experiment participants

Two hundred and thirty participants (93 females, 136 males, one prefer not to say; mean age = 25.95 years, SD = 9.63 years) conducted the rating experiment. This ensured that a minimum of ten participants rated each action on one of 23 different action characteristics; participants were randomly assigned to rate the different characteristics (see OSM for demographic information detail). This decision was made following the methods used in Sutherland et al. (2013), in which a minimum of six participants rated each of the stimuli for each characteristic. Participants were either recruited via Prolific (n = 206) and compensated £3, via the internal University of York SONA system (n = 9) and compensated with course credit, or recruited opportunistically through social media (n = 15). All participants had normal or corrected-to-normal vision. This research project was approved by the ethics committee of the Department of Psychology, University of York, and was performed in accordance with the ethical standards laid down in the 1964 Declaration of Helsinki.

### Experimental procedure

**Fig. 3:**
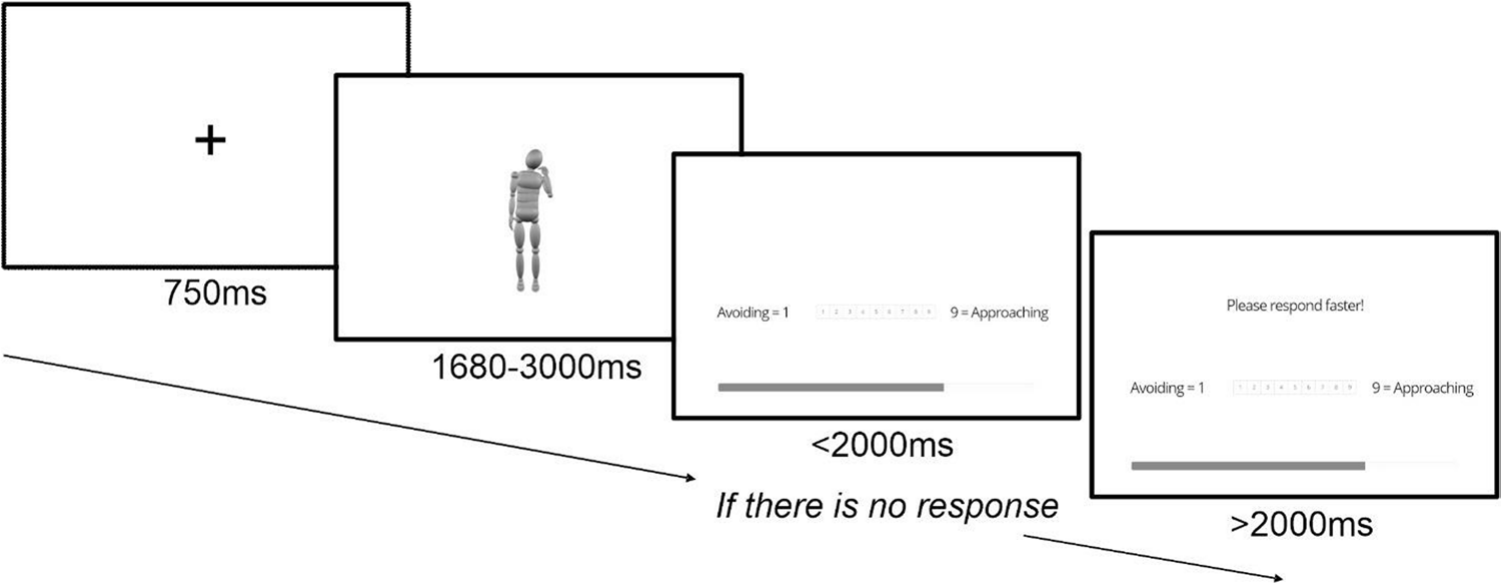
Standard trial structure for the rating of all 23 characteristics. Illustrated is a trial for the “Avoiding-Approaching” characteristic. Following presentation of the action, response buttons are presented on screen, along with an experimental progress bar

For an exploratory factor analysis, a 10:1 participant-to-variable ratio is considered appropriate (Howard, 2016; Kyriazos, 2018). Within our experimental design this ratio equates to the action-to-characteristic ratio, and thus the experimental design consisted of 240 actions (items) each rated on the 23 different action characteristics to ensure an adequate sample size. 230 participants each viewed all 240 actions rating them on one of the different characteristics, where ten participants were allocated to each characteristic. The experiment was implemented via the Gorilla Experiment Builder (Anwyl-Irvine et al., 2019; Anwyl-Irvine et al., 2021). Once participants entered the experiment site through an internet browser on either a laptop or desktop computer, participants completed a consent form and entered simple demographic information (age and gender). Instructions on the experimental task were then displayed, including which of the 23 action characteristics the participant was going to evaluate the actions on. Before the experiment itself, participants took part in a set of eight practice trials identical to those used during the experiment. On each trial, participants viewed first a 750-ms fixation cross, then the video of the action for its duration, and finally a response screen showing a 1–9 Likert scale where the participant had to indicate their immediate evaluation of the action by clicking an on-screen button with the mouse. Once a response was registered, the next trial commenced. If participants failed to respond within 2 s of the end of the action, a prompt “Please respond quickly” appeared at the top of the response screen to encourage quick first impressions of the actions (see Fig. 3). Following completion of the practice trials, participants began the experiment itself. The experiment consisted of 244 trials in total, 240 trials where each of the 240 different actions were shown, and an additional four catch trials. During catch trials the response screen explicitly asked the participant to press a specific button; these were included to assess participant attention during the experiment. The experiment was divided into blocks of 61 trials, where participants were allowed a break in between blocks of up to 1 min, to help maintain concentration throughout the duration of the task. A progress bar was presented on-screen along with the response buttons to provide participants with an indication of how far through the experiment they were; on average the experiment took approximately 30 min to complete the task.

### Data analysis

Data were analysed using the ‘irr’ (Gamer et al., 2019), ‘psych’ (Revelle, 2022), ‘performance’ (Lüdecke et al., 2021),‘datawizard’ (Makowski et al., 2021), ‘parameters’ (Lüdecke et al., 2020), and ‘lavaan’ (Rosseel, 2012) packages in R Studio (R Core Team, 2020). Ratings of each of the 240 actions on each characteristic were averaged across each group of ten participants. An exploratory factor analysis (EFA) was first selected to analyse the data because the research aim was exploratory and there were no strong prior hypotheses as to which factors underlie the perception of actions (Schmitt, 2011). The final interpretation of the model considered the context that the analysis was exploratory, and these factors were likely to be correlated (Fabrigar et al., 1999; Schmitt, 2011). Once suitable EFA models were generated, we ran confirmatory factor analysis conversions. This was done by randomly partitioning the data into 50% model and test proportions and running an EFA analysis on the 50% model proportion, then converting this model to a CFA using the 50% test proportion. The relative goodness of fit of competing CFA models were assessed to establish the best fitting CFA model, and by extension the best fitting EFA model for the data.

## Results

Five participants were tested and subsequently removed from the data analysis and replaced due to poor data quality. This was determined as participants who met two of the following three criteria: > 1 min to respond to one of the trials, and > 3 s to respond in over 10% of trials. For the exploratory factor analysis, for each of the 23 characteristics a mean average of the ten participants ratings of the 240 different actions was used. One participant had an incomplete trial, as such one action’s average rating for one of the characteristics was based on nine participants ratings instead of ten.

### Reliability

The intra-class correlation coefficient (Koo & Li, 2016) was calculated for each of the 23 characteristics to assess the consistency of ratings between the ten participants. A two-way model with “average” unit of ratings was used for each of these analyses, the results of which are presented in Table 2. Two characteristics were found to have poor inter-rater reliability (κ<.5), ten were moderate (.5<κ<.75), ten were good (.75<κ< .9), and one was excellent (κ>.9; see Table 2). The two characteristics with poor inter-rater reliability (Breaking-Making and Removing-Adding) were removed from all subsequent analyses (Koo & Li, 2016).

**Table 2:** Inter-rater reliability for each group of ten participants rating actions on one of the 23 characteristics

Characteristic	kappa	95% Confidence interval	F-statistic	Interpretation
Accidental - Intentional	.869	.843 < κ < .893	F(239,2151) = 7.65 , p<.001	Good
Angry - Happy	.915	.898 < κ < .93	F(239,2151) = 11.8 , p<.001	Excellent
Anti-Social - Pro-Social	.74	.689 < κ < .787	F(239,2151) = 3.85 , p<.001	Moderate
Anxious - Confident	.802	.762 < κ < .838	F(239,2151) = 5.37 , p.<001	Good
Approaching - Avoiding	.727	.671 < κ < .777	F(239,2151) = 3.94 , p<.001	Moderate
Breaking - Making	.485	.385 < κ < .575	F(239,2151) = 2 , p<.001	Poor
Disapproving – Approving	.752	.681 < κ < .807	F(239,2151) = 5.1 , p<.001	Good
Hesitant - Fluent	.728	.673 < κ < .777	F(239,2151) = 3.84 , p<.001	Moderate
Hiding - Uncovering	.717	.669 < κ < .773	F(239,2151) = 3.62 , p<.001	Moderate
Ignoring - Communicating	.835	.798 < κ < .867	F(239,2151) = 6.81 , p<.001	Good
Ingesting - Expelling	.521	.428 < κ < .605	F(239,2151) = 2.16 , p<.001	Moderate
Lowering - Raising	.796	.744 < κ < .839	F(239,2151) = 5.87 , p<.001	Good
Low-Speed - High-Speed	.773	.705 < κ < .825	F(239,2151) = 5.64 , p<.001	Good
Pulling - Pushing	.787	.744 < κ < .825	F(239,2151) = 4.9 , p<.001	Good
Rejecting - Desiring	.783	.739 < κ < .823	F(239,2151) = 4.92 , p<.001	Good
Releasing - Getting	.566	.480 < κ < .643	F(239,2151) = 2.35 , p<.001	Moderate
Removing - Adding	.424	.310 < κ < .527	F(239,2151) = 1.74 , p<.001	Poor
Straightening - Bending	.601	.513 < κ < .677	F(239,2151) = 2.88 , p<.001	Moderate
Subordinate - Dominant	.738	.686 < κ < .785	F(239,2151) = 3.94 , p<.001	Moderate
Threatening - Protecting	.821	.783 < κ < .855	F(239,2151) = 6.08 , p<.001	Good
Uncontrolled - Controlled	.642	.566 < κ < .709	F(239,2151) = 3.13 , p<.001	Moderate
Untrustworthy - Trustworthy	.617	.532 < κ < .690	F(239,2151) = 2.99 , p<.001	Moderate
Weak - Powerful	.846	.812 < κ < .876	F(239,2151) = 7.25 , p<.001	Good

The averaged ratings of the actions for each of the remaining 21 characteristics were normally distributed when plotted on a histogram. The Kaiser-Meyer-Olkin criterion (Dziuban & Shirkey, 1974; Kaiser, 1974) for each of the 21 characteristics indicated that one of the characteristics – ’Straightening-Bending‘ – had a measure of sampling adequacy (MSA) value below .6 (.503), and so did not psychometrically relate to the rest of the data. The other 20 characteristics had MSA values ranging from .641 (‘mediocre’) to .94 (‘marvellous’; see Table 3). Thus, the characteristic of ’Straightening-Bending’ was removed from all subsequent analyses. For the remaining 20 characteristics, the overall KMO MSA value was .88 (‘meritorious’).

**Table 3:** Measures of sampling adequacy for action characteristics

Characteristic	MSA
Accidental - Intentional	.838
Angry - Happy	.884
Anti-Social - Pro-Social	.914
Anxious - Confident	.876
Approaching - Avoiding	.94
Disapproving - Approving	.893
Hesitant - Fluent	.865
Hiding - Uncovering	.924
Ignoring - Communicating	.836
Ingesting - Expelling	.878
Lowering - Raising	.908
Low-Speed - High-Speed	.853
Pulling - Pushing	.854
Rejecting - Desiring	.909
Releasing - Getting	.641
Straightening - Bending	.503
Subordinate - Dominant	.888
Threatening - Protecting	.853
Uncontrolled - Controlled	.725
Untrustworthy - Trustworthy	.922
Weak - Powerful	.893

**Fig. 4:**
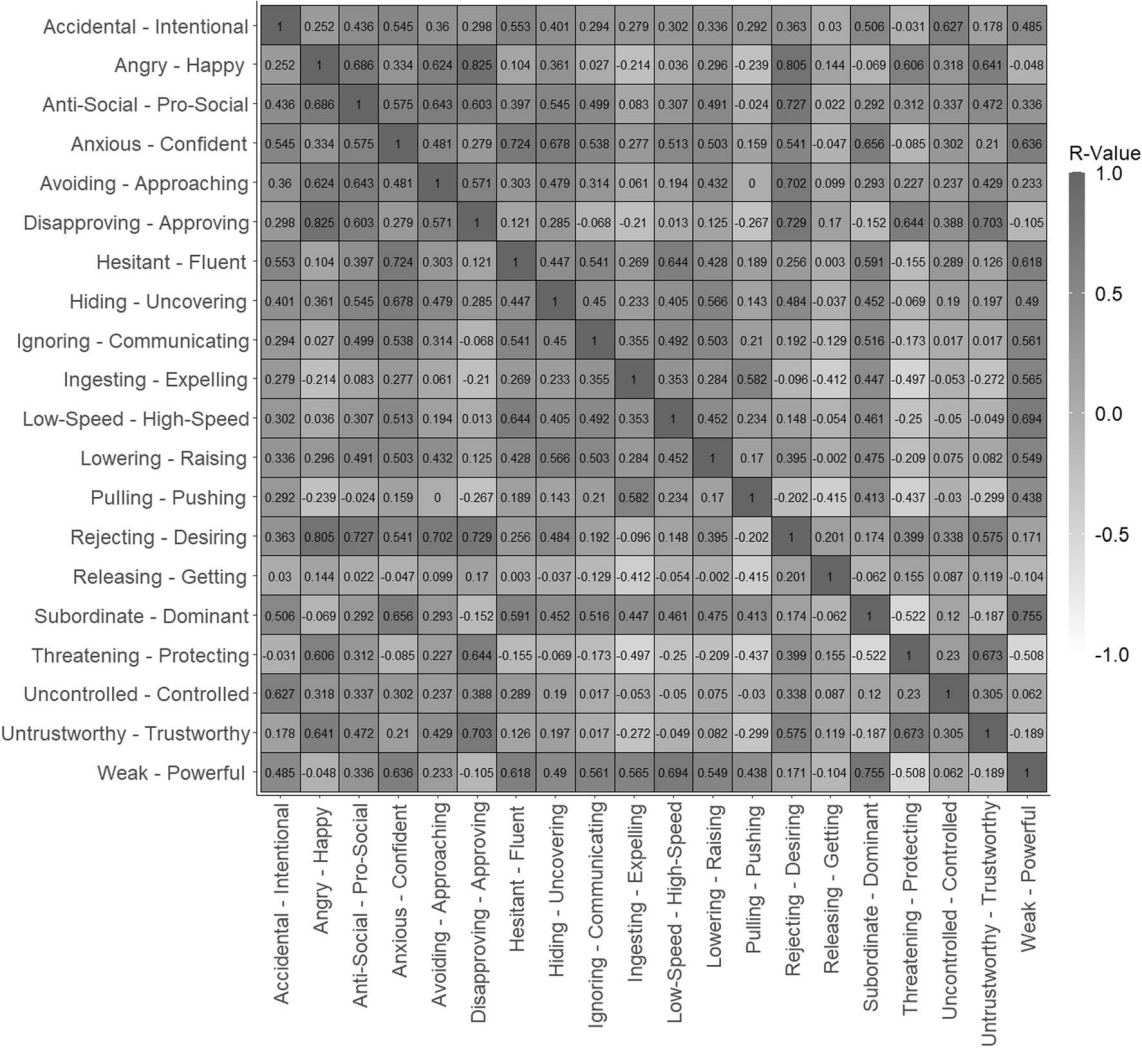
Raw Pearson’s R correlation matrix between the 20 retained characteristics

Furthermore, Bartletts test of sphericity was significant (χ^2^(190) = 3637.72, p < .001). These measures both indicated that the characteristics were sufficiently psychometrically related for an exploratory factor analysis to be conducted. The raw Pearson’s R correlation matrix between the retained 20 characteristics is presented in Fig. 4.

### Determining the model

**Fig. 5:**
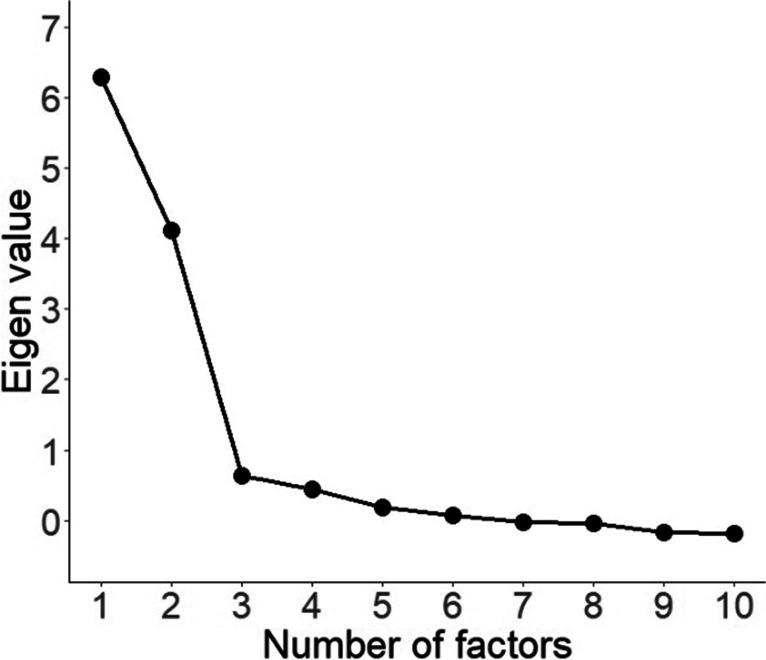
Scree plot of Eigen values for exploratory factor analysis models up to ten factors

Two methods were used to determine the number of factors to extract (Fabrigar et al., 1999; Howard, 2016): first a visual scree plot analysis (Zoski & Jurs, 1990), and second a Parallel Analysis (Patil et al., 2008, 2017). These methods were selected because parallel analysis is generally considered to be one of the more robust methods, and although visual scree plot analyses can be variable, it is an intuitive and generally accurate method (Zwick & Velicer, 1986). Visual analysis of the scree plot (see Fig. 5) indicated that although two latent factors are distinctively above the break or ‘elbow’ (Howard, 2016), there is a small second drop in Eigen values after the third and fourth factors, which indicates that these may also be considered additional ‘non-trivial factors’ (Zoski & Jurs, 1990). In agreement with this later interpretation, the Parallel Analysis (Patil et al., 2008, 2017) indicated that four factors should be extracted.

Initially a four-factor principal axis factoring (PAF) solution (Howard, 2016) was run and an oblique rotation of ’direct oblimin‘ was applied to allow for correlations between the factors (Costello & Osborne, 2005; Fabrigar et al., 1999 ; Schmitt, 2011). A PAF was used as it gives accurate results with a lower number of assumptions than maximum likelihood (Howard, 2016), and gives more accurate results than principal component analysis (PCA) if communalities are low (Kahn, 2006). Furthermore, PAF is preferable when determining the latent factors underlying a potentially non-normally distributed dataset, as was the case here. We rotated the model to improve fit (Schmitt, 2011), selecting an oblique rotation to allow for correlations. This was because it was anticipated that the dimensions would be correlated, as generally this is the case with psychological factors (Fabrigar et al., 1999; Schmitt, 2011). In addition, an oblique rotation method can produce both correlated and uncorrelated factors, and so if the factors are uncorrelated an oblique rotation method would still produce reliable results. Whereas an orthogonal rotation method forces uncorrelated factors, which, if the factors are to any degree correlated, would produce a less accurate model with potentially inflated item loadings for would-be correlated factors (Costello & Osborne, 2005; Schmitt, 2011).

The subsequent structure matrix of the four-factor model (Table 4) was interpreted following a more conservative .50–.30 interpretation of Howard's (2016) .40–.30–.20 rule. This was based upon the suggestion by Tabachnick and Fidell (2007) that the primary loading should be above .45, instead of .4 (Howard, 2016). Hence, in this experiment, characteristics were considered as substantial loadings if they had a primary loading above .5 and cross loadings below .3. Our four-factor model accounted for 59.9% of the variance in the data. For clarity we named the factors without using any of the words used to define the action characteristics (Reio Jr & Shuck, 2015).

**Table 4:** Correlations between characteristics and latent factors. Communality values represent the amount of variance in each characteristic that is accounted for by the model

Characteristic	Factor 1	Factor 2	Factor 3	Factor 4	Communality
Accidental - Intentional	.212	.056	**.775**	.143	.831
Angry - Happy	-.051	**.926**	-.005	-.008	.848
Anti-Social - Pro-Social	.342	.**736**	.017	.087	.745
Anxious - Confident	**.703**	.251	.186	-.009	.745
Avoiding - Approaching	.27	**.672**	-.008	.073	.570
Disapproving - Approving	-.174	**.837**	.167	-.034	.810
Hesitant - Fluent	**.722**	-.043	.289	-.121	.641
Hiding - Uncovering	.544	.381	-.012	.094	.530
Ignoring - Communicating	**.656**	.117	-.112	.101	.489
Ingesting - Expelling	.144	-.019	.007	**.725**	.646
Lowering - Raising	**.626**	.284	-.133	.084	.509
Low-Speed - High-Speed	**.714**	-.008	-.07	.011	.491
Pulling - Pushing	-.04	-.092	.132	**.756**	.611
Rejecting - Desiring	.247	**.781**	.038	-.105	.784
Releasing - Getting	.234	-.094	.079	**-.651**	.323
Subordinate - Dominant	**.722**	-.17	.181	.117	.702
Threatening - Protecting	-.434	.629	.059	-.205	.701
Uncontrolled - Controlled	-.116	.116	**.757**	-.055	.608
Untrustworthy - Trustworthy	-.143	**.687**	.104	-.121	.583
Weak - Powerful	**.789**	-.108	.067	.19	.818

*Note:* Substantial loadings are highlighted in bold and defined using an adjusted, more conservative, interpretation of Howard's (2016) .40–.30–.20 rule, with primary loadings above .5 and cross loadings below .3

The first factor, ‘Feeble-Formidable’, accounted for 22.1% of variance. ‘Feeble’ represented, in descending order of influence, the substantial loadings of weak, subordinate, hesitant, low-speed, anxious, ignoring, and lowering. Whilst ’Formidable” represented, in descending order of influence, the substantial loadings of powerful, dominant, fluent, high-speed, confident, communicating, and raising. The second factor, ‘Unfriendly-Friendly’, accounted for 22% of the variance. ‘Unfriendly’ predominantly represented the substantial loadings of angry, disapproving and rejecting, but also untrustworthy and avoiding. Whilst ‘Friendly’ predominantly represented the substantial loadings of happy, approving and desiring, but also trustworthy and approaching. The third factor, ‘Unplanned-Planned’, accounted for 7.2% of the variance, a smaller proportion than the first two. ‘Unplanned’ represented the substantial loadings of accidental and uncontrolled. Whilst ‘Planned’ represented the substantial loadings of intentional and controlled. The fourth factor, ‘Adduction-Abduction’, accounted for a similarly small proportion of the variance of 8.6%. ‘Adduction’ represented the substantial loadings of pulling, ingesting, and getting, which were considered to reflect movement towards the trunk of the body or away from the observer. ‘Abduction’, in contrast, represented the substantial loadings of pushing, expelling, and releasing, which reflect movement away from the trunk of the body or towards the observer. Within-factor correlations are illustrated in Table 5.

**Table 5:** Between-factor correlation matrix of r values for the four-factor model

	Feeble - Formidable	Unfriendly - Friendly	Unplanned - Planned	Adduction - Abduction
Feeble - Formidable				
Unfriendly - Friendly	.15			
Unplanned – Planned	.29	.32		
Adduction – Abduction	.44	-.26	.06	

Due to the first two factors accounting for a much larger proportion of the variance, higher Eigenvalues, and are more overdetermined with a larger number of substantially loading characteristics than the third and fourth factors, we regard to the model having 2 + 2 factors. With ‘Feeble-Formidable’ (Eigenvalue = 6.531) and ‘Unfriendly-Friendly’ (Eigenvalue = 4.598) being the more influential factors, whilst ‘Unplanned-Planned’ (Eigenvalue = 1.015) and ‘Adduction-Abduction’ (Eigenvalue = 0.84) being the less influential factors.

### Testing model fit

As the first two factors explained a much larger proportion of the variance in the data than the second two factors, we ran model comparisons of the competing two- and four-factor models to confirm whether a four-factor model was a more appropriate fit for the data than a two-factor model. This is particularly important as the third extracted factor (Planned-Unplanned) may be considered a less robust or unstable factor because it has fewer than three substantially loading characteristics and so it is less overdetermined (Costello & Osborne, 2005; Hogarty et al., 2005). As such a two-dimensional model, which does not include this potentially unstable factor, may be a more reliable representation of the data. To verify that a two- or four-factor model is the most suitable, we also included in the model comparison the three- and five-factor models. We anticipated that oblique models, rather than orthogonal models, would be more appropriate for the representation of action perception, in line with the general pattern seen in psychological phenomena (Fabrigar et al., 1999; Schmitt, 2011) and the between-factor correlations (see Table 5) corroborate this approach. As such, we compared the two-, three-, four- and five-factor models with oblique rotations. Model comparisons were run by converting the EFA models of interest into confirmatory factor analyses (CFA) to assess and compare the relative goodness-of-fit of the competing models of interest. This was necessary as to allow the EFA models to be truly exploratory (Schmitt, 2011) the PAF factor extraction method was selected, however PAF solutions do not allow for goodness-of fit indices (Howard, 2016).

In this analysis, two-, three-, four-, and five-factor EFA models were extracted using PAF with oblique rotations. These four EFA models were converted to CFA models by randomly partitioning the data into 50% model and test proportions, with 120 actions randomly allocated to the model proportion and 120 actions allocated to the test proportion. An EFA analysis was run on the 50% model proportion, then this was converted to a CFA using the 50% test proportion. This process was simulated 200 times as the goodness-of-fit statistics for each model were dependent upon the random partitioning of the data into the 50% model and 50% test proportions during each simulation, and 200 samples were required for Akaike information criterion (AIC; Akaike, 1974) values to be reliable (Hooper et al., 2008). Following similar methods to those used by Sutherland et al. (2013), the mean average and 95% confidence intervals of a number of goodness-of-fit statistics were compared (see Table 6), including: the model χ^2^ (Hooper et al., 2008), Confirmatory Fit Index (CFI; Hu & Bentler, 1999), root mean square error of approximation (RMSEA; Hooper et al., 2008) and AIC (Hooper et al., 2008).

**Table 6:** Means and 95% confidence intervals of the goodness-of-fit statistics from the 200 simulations of the CFA models with two-, three-, four- or five factors

Model	χ2 (df), p-value [95% CI of χ2]	RMSEA, p-value [95% CI of RMSEA]	CFI [95% CI]	AIC [95% CI]
Two factors	χ2(169) = 809.581, p<.001 [803.685, 815.477]	.178, p<.001 [.177, .178]	.647 [.644, .649]	6224 [6212, 6235]
Three factors	χ2(167) = 767.512, p<.001 [760.631, 774.392]	.173, p<.001 [.172, .174]	.669 [.666, .672]	6185 [6174, 6197]
Four factors	χ2(164) = 690.969, p<.001 [683.219, 698.718]	.163, p<.001 [.162, .165]	.709 [.705, .713]	6115 [6103, 6127]
Five factors	χ2(160) = 736.496, p<.001 [727.677, 745.316]	.173, p<.001 [.172, .174]	.682 [.678, .686]	6168 [6157, 6180]

Whether the average goodness-of-fit statistics for each of the converted CFA models met the traditional thresholds of acceptability was not considered, for a number of reasons. First the CFA model goodness-of-fit statistics for each simulation was dependent upon the random partitioning of the data, and which 120 actions were allocated to the model and test proportions. Thus, the average CFA goodness-of-fit statistics are not direct measures of the original goodness-of-fit of the EFA models. Secondly, there is some debate about the implementation and interpretation of traditional goodness-of-fit statistics as the statistics, such as chi-square, RMSEA and CFI, can be sensitive to sample size and potentially non-normally distributed data, as is the case with the current data set, (Dogan et al., 2015; Kyriazos, 2018) and the thresholds for statistics may be arbitrary (Lai & Green, 2016). Thirdly, the interpretation of the AIC values is limited to which model has the relatively lowest value, as this indicates the best fitting model, because the absolute AIC value is not indicative of how well the model fits the data (Akaike, 1974). For these reasons, and because the aim of this EFA to CFA conversion analysis was to distinguish which of the respective EFA models was comparatively the better fit to the data, whether the CFA models average goodness-of-fit statistics met the traditional thresholds was not considered. Instead, the analysis focused on which CFA model had the better average goodness-of-fit statistics, as this indicated which of the respective original EFA models was the better fit for the data. The average goodness-of-fit statistics from the 200 simulations of the four different models are presented in Table 6.

The four-factor model was a better fit than the two-factor model, as indicated by the lower mean AIC, RMSEA and model χ^2^, and higher CFI. To verify whether the four-factor model was optimal and was independent of the actions used during the experiment and remained robust with data with more variation, we progressively decimated our data by randomly removing actions and reducing the 12:1 action to characteristic ratio (240 actions:20 characteristics) down to 5:1. In this analysis we ran 200 simulations of the two-, three-, four- and five-factor models for every ten-action decrement from 240 to 100 actions. As such this analysis represents 3,000 simulations of EFA to CFA conversions for each of the two-, three-, four- and five-factor oblique models (12,000 in total). For each ten-action increment the actions that were removed were randomly selected and different for each of the 200 simulations.

**Fig. 6:**
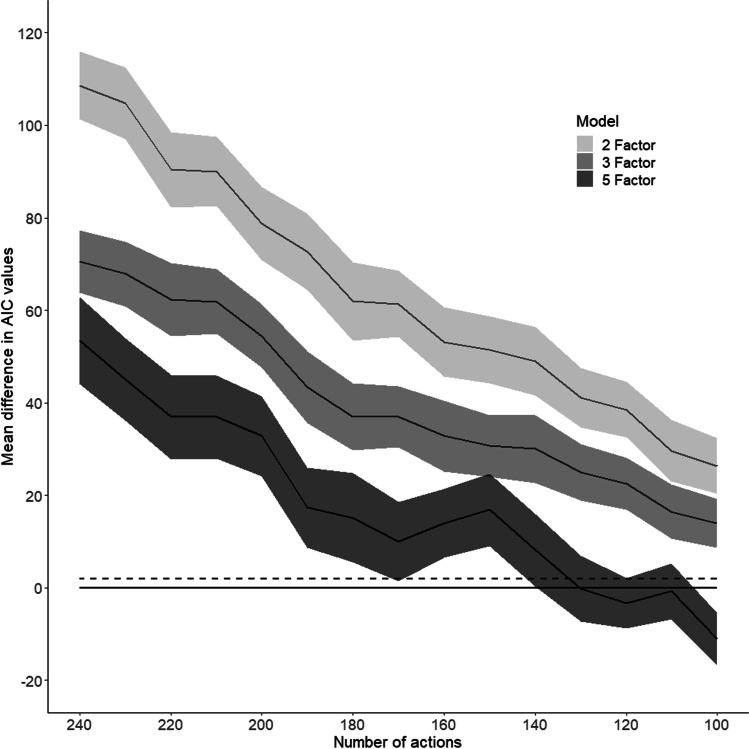
Model comparisons following decimation by the number of actions. Mean difference in AIC values (and 95% confidence intervals – grey regions) between the two-, three- and five-factor models for 240 actions down to 100 actions. The solid line at 0 represents the four-factor model; the dashed line represents a difference in AIC value of 2. A difference in AIC of 2 or above is generally considered to represent a practical difference between models (Cavanaugh & Neath, 2019)

As AIC values will vary depending on the number of actions included in the models, a direct comparison between the AIC values of models from different ten-action increments was not possible. As such the difference in AIC values between the two-, three- and five-factor models to the four-factor model were calculated for each simulation instead. These differences were calculated by subtracting the AIC value of the four-factor model from the AIC value of the other models. From the 200 simulations mean AIC difference and 95% confidence intervals were calculated for the two-, three- and five-factor model. These model comparisons are represented in Fig. 6. Here, a positive AIC difference for the other models indicates that the four-factor model is more optimal, whilst negative differences indicate that the alternate model is optimal.

As shown in Fig. 6, the four-factor model remains the most appropriate fit for the decimated data from 240 actions (representing a 12:1 action to characteristic ratio) down to 140 actions (representing a 7:1 action to characteristic ratio). From 140 to 110 actions (11:2 action to characteristic ratio) either the four- or five-factor model could be the most appropriate as represented by the 95% confidence interval range of the five-factor model overlapping with the four-factor model (solid line). By 100 actions (5:1 action to characteristic ratio) the five-factor model becomes the most appropriate fit for the data; this is likely due to the higher number of factors explaining a larger proportion of the variance, over the parsimony of the four-factor model. Importantly, the characteristics that represent the factors in the two-, three-, four- and five-factor models were consistent for all ten-action interval decimations, with the factors we named Feeble-Formidable and Unfriendly-Friendly being extracted in the two-factor model, Unplanned-Planned was additionally extracted in the three-factor model, and Abducting-Adducting additionally extracted in the four-factor model. In the five-factor model the fifth factor represented dominant, protecting, and trustworthy characteristics.

In summary, by decimating our data by running multiple CFA to EFA simulations with randomly removed actions and decreasing the action to characteristic ratio demonstrates that from a 12:1 to 7:1 action to characteristic ratio the four-factor model has the best fit. As noted in (Howard, 2016) there is some degree of variation in the recommended subject to item ratio (equivalently here action to characteristic ratio), although a ratio of 10:1 is regarded as appropriate (Howard, 2016; Kyriazos, 2018). This analysis shows that the optimal EFA solution remains consistent and robust, however, once non-optimal action to characteristic ratios of 5:1 are reached a different optimal solution may emerge, however, this would be less parsimonious.

**Fig. 7:**
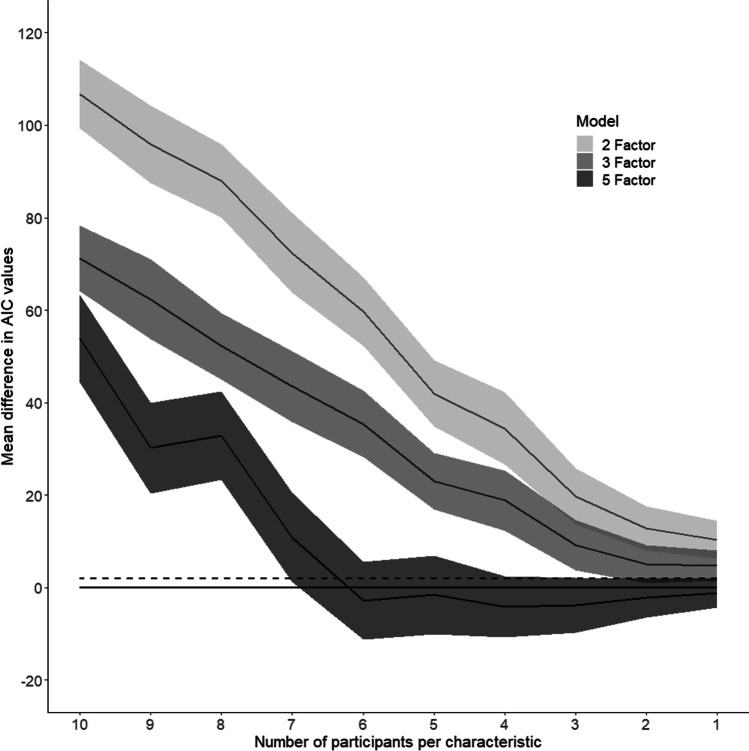
Model comparisons following decimation of the number of participants contributing to characteristic ratings. Mean difference in AIC values (and 95% confidence intervals – grey regions) between the four-factor models and the two-, three- and five-factor models for average characteristic ratings calculated from 10 participants down to 1. The solid line at 0 represents the four-factor model; the dashed line represents a difference in AIC value of 2. A difference in AIC of 2 or above is generally considered to represent a practical difference between models (Cavanaugh & Neath, 2019)

We also examined whether the four-factor model would remain robust when fewer participants contributed to the average characteristic ratings of each action, and to explore the minimum number of participants needed for the four-factor model to emerge as the most appropriate, we decimated the data by the number of participants included. In the experiment ratings for each characteristic and action were calculated by averaging across ten participants. For this analysis 200 EFA-CFA conversions were simulated on datasets with average ratings calculated from between ten and one participants, totalling 2,000 simulations of each model type. Participants were randomly selected to be included in each simulation. For the two-, three- and five-factor models mean difference in AIC values in comparison to the four-factor model and 95% confidence intervals were calculated (see Fig. 7).

The simulated two-, three-, four- and five-factor models generated from data by participants retained the same dimensions and similar characteristic loadings to the non-decimated data. For simulations using data when average characteristic ratings are calculated from ten to seven participants, the four-factor model remains the best fit compared to the two-, three- and five-factor models. Figure 7 also demonstrates that when reducing the number of contributing participants to the average characteristic ratings differences in AIC values of the two-, three-, four- and five-factor models become less distinct, likely resulting from the models with fewer participants becoming less good fits to the data. For those simulations with data from six or fewer participants, either the four-factor or the five-factor model could be considered to be appropriate fits. Delineating the two models is not possible due to overlapping confidence intervals, although the five-factor model explains a little more of the variance probably due to the higher number of factors retained. In summary, the four-factor model remains robust and is the best fit for average action characteristics calculated from seven or more participants; however, if fewer participants are tested the relative appropriateness of the different models become less distinct, and thus it becomes harder to delineate the optimal model.

**Fig. 8:**
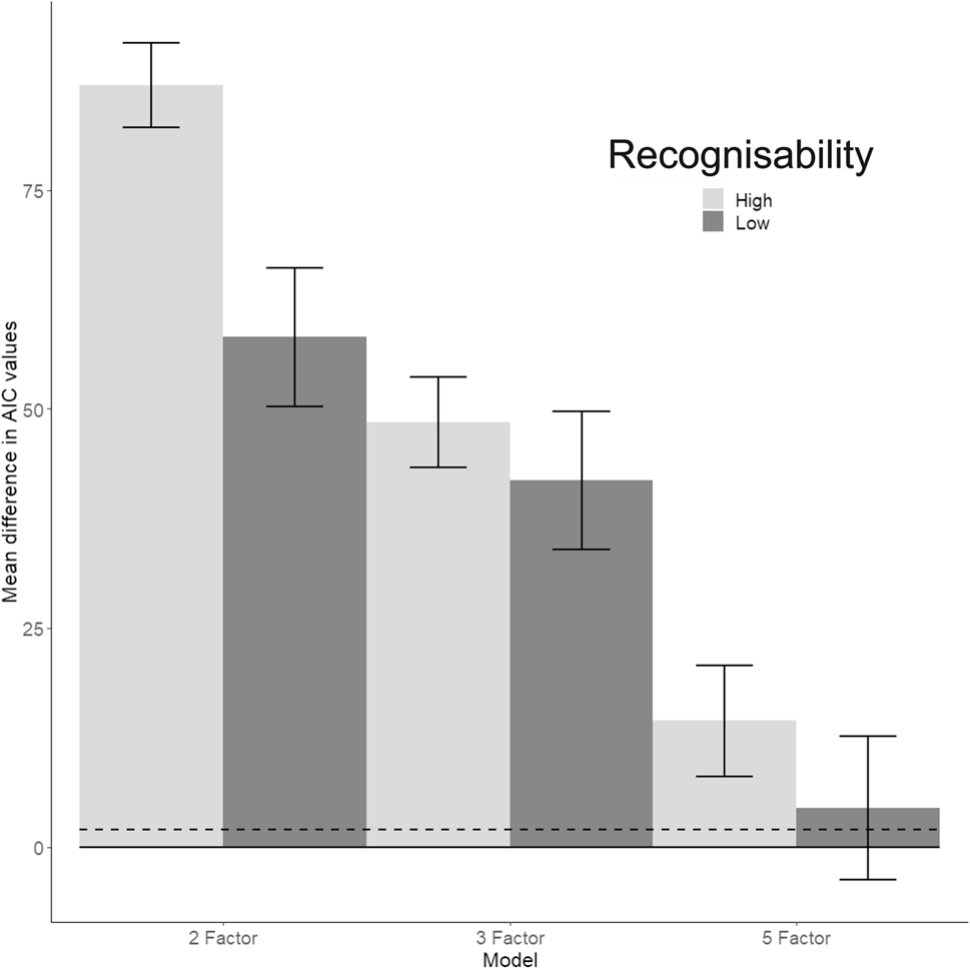
Model comparisons for actions with high and low recognisability. Mean difference in AIC values (and 95% confidence interval error bars) between the two-, three- and five-factor models compared to the four-factor models. The solid line at 0 represents the respective four-factor model; the dashed line represents a difference in AIC value of 2. A difference in AIC of 2 or above is generally considered to represent a practical difference between models (Cavanaugh & Neath, 2019)

Our test of action recognition indicated that an independent group of participants were able to correctly identify the action goal or actor intention for most of our actions (Fig. 2); however, some of our actions were difficult to correctly identify. Inability to correctly identify some actions may have had an impact on ratings of their characteristics, and potentially on the optimal model of action space. To test how action recognisability influenced model fit, and whether the four-factor model remained optimal, we separated actions into those that were best recognised and those that were less well recognised, and used similar methods to those described above to compare models. Our previous analysis showed that the minimum number of actions for the four-factor model to remain robust must be greater than 140 (representing a 7:1 action to characteristic ratio). In order to generate datasets with enough actions to delineate the optimal model we analysed data from the 180 actions that were most recognisable and data from the 180 actions that were least recognisable (consequently data from 60 actions contributed to both data sets). For each dataset we simulated 200 EFA-CFA conversions with 50% test and training proportions. The mean and 95% confidence intervals of the difference in AIC values between the two-, three- and five-factor models and the respective four-factor model were calculated (illustrated in Fig. 8).

In comparison to the two-, three- and five-factor models, the four-factor model remained the best fit to the data irrespective of whether the data were sourced from the most recognisable or least recognisable actions; most 95% confidence intervals calculated for the two-, three- and five-factor models did not overlap with the 2 AIC points of the four-factor model (Fig. 8). There was some overlap for the lower bound of the 95% confidence interval for the five-factor model based upon the least recognisable actions with the 2 AIC thresholds for the four-factor model. Although the four-factor model appears to be the best fit, either the four- or five-factor models could be appropriate when action recognisability is low. Importantly, there was little variation in mean AIC value, and confidence intervals overlapped, when comparing each of the two-, three- and five-factor models calculated from the most recognisable and least recognisable actions. Furthermore, the characteristics that load onto the factors in the two-, three-, four- and five-factor models were similar for both groups of actions, and the original four-factor model. This shows that the four-factor model is optimal irrespective of whether the actions are well recognised or not; however, the robustness of the optimal four-factor models is less distinct for less recognisable actions.

To verify the reliability of the factors in the selected four-factor model with oblique rotation, two-way intra-class correlation coefficients with ‘average’ unit of ratings were run to assess the consistency within the average ratings of the characteristics that substantially loaded onto each factor were run (Koo & Li, 2016, see Table 7). For the purposes of this analysis the average ratings for the negative substantial loading “Releasing-Getting” characteristic for the Adduction–Abduction factor were reversed, so that the characteristic represented “Getting-Releasing” and all substantial loadings for this factor were in the same direction. All substantially loading characteristics for the other factors were positive loadings and so did not require this adjustment.

**Table 7:** Inter-rater reliability for the four factors within the substantially loading characteristics

Characteristic	kappa	95% Confidence Interval	F-Statistic	Interpretation
Feeble - Formidable	.896	[.875, .915]	F(239,1434) = 9.65 , p<.001	Good
Unfriendly - Friendly	.907	[.888, .925]	F(239,956) = 10.8 , p<.001	Excellent
Unplanned - Planned	.755	[.684, .81]	F(239,239) = 4.08 , p<.001	Good
Adduction - Abduction	.703	[.631, .762]	F(239,478) = 3.36 , p<.001	Moderate

The results of the intraclass correlation coefficient show that all four factors demonstrate an acceptable degree of inter-characteristic reliability (Koo & Li, 2016). In line with the 2 + 2 structure of the four-dimension model with oblique rotation, the two more influential factors, Feeble-Formidable and Unfriendly-Friendly, had notably higher intraclass correlation coefficients than the two less influential factors, Unplanned-Planned and Adduction-Abduction.

### Response times

Although this experiment did not explicitly aim to analyse response times, an interesting effect was observed whilst calculating the average response time for each characteristic (see Table 8). Across all actions for all characteristics the average response time to trials was 1,125 ms (SD = 586 ms). However, there was a broad grouping of the average speed of response times for each characteristic by the factors the characteristics load onto. All characteristics that substantially loaded onto the Unfriendly-Friendly factor had the fastest average response times (range 946–1,059 ms). In contrast, the characteristics that substantially load onto the Feeble-Formidable factor had longer response times, between 1,108 ms and 1,157 ms. Even longer response times were seen for the two factors that substantially loaded onto Unplanned-Planned, between 1,196 ms and 1,206 ms. Whilst the characteristics that load onto Adduction-Abduction, did not appear to show any particular pattern in response times.

**Table 8:** Average response times for each characteristic in order of fastest to slowest and the factor the characteristic substantially loads onto

Characteristic	Factor	Mean	SD
**Angry - Happy**	**Unfriendly - Friendly**	**946.22**	**399.33**
Rejecting - Desiring	Unfriendly - Friendly	1001.73	607.53
Disapproving - Approving	Unfriendly - Friendly	1032.05	410.97
Untrustworthy – Trustworthy	Unfriendly - Friendly	1032.05	517.66
Approaching - Avoiding	Unfriendly - Friendly	1058.70	517.57
Threatening - Protecting		1059.03	415.62
**Ingesting - Expelling**	**Adduction - Abduction**	**1067.99**	**1000.91**
Anxious - Confident	Feeble - Formidable	1108.37	409.41
Lowering - Raising	Feeble - Formidable	1110.36	438.04
Hesitant - Fluent	Feeble - Formidable	1125.69	540.94
**Weak - Powerful**	**Feeble - Formidable**	**1133.51**	**551.7941**
Low-Speed - High-Speed	Feeble - Formidable	1141.61	553.98
Subordinate - Dominant	Feeble - Formidable	1157.41	473.21
Pulling - Pushing	Adduction - Abduction	**1188.24**	**640.76**
Uncontrolled - Controlled	Unplanned - Planned	1196.48	484.42
**Accidental - Intentional**	**Unplanned - Planned**	**1206.17**	**488.95**
Releasing - Getting	Adduction - Abduction	1210.15	479.18
Anti-Social - Pro-Social		1223.61	560.34
Ignoring - Communicating	Feeble - Formidable	1238.30	603.38
Hiding - Uncovering		1313.91	637.52

*Note.* The characteristic that loads most substantially onto each factor is highlighted in bold. Blank rows indicate that the characteristic had no primary substantial loading onto a factor

To explore this pattern further a one-way independent measures analysis of variance (ANOVA) was conducted in IBM SPSS 28. This analysis examined if participants’ average response times to the 240 actions differed depending upon which of the four factors the rated characteristic loaded onto. Here, 70 participants’ average ratings were allocated to the Feeble-Formidable group (ten participants for each of the substantially loading seven characteristics; mean = 1,145 ms, SD = 25 ms), 50 participants’ average ratings were allocated to the Unfriendly-Friendly group (mean = 1,014 ms, SD = 25,ms), 20 participants’ average ratings to the Unplanned-Planned group (mean = 1,201 ms, SD = 51 ms), and 30 participants’ average ratings to the Adduction-Abduction group (mean = 1,155 ms, SD = 44 ms). Shapiro-Wilk tests of normality showed that participants’ average response times were normally distributed for all four factors (Feeble-Formidable = W(70) = .966, p = .052; Unfriendly-Friendly = W(50) = .978, p = .484, Unplanned-Planned = W(20) = .913, p = .074; Adduction-Abduction = W(30) = .978, p = .757). A Levene’s test for homogeneity of variance was non-significant (F(3,166) = 1.08, p = .361), indicating equal variances between the factors.

The main effect of factor was significant (F(3,166) = 5.92, p < .001), indicating that there were significant differences in participants’ average response times depending upon which of the four factors the rated characteristic loaded onto. Hochberg’s GT2 post hoc tests were selected for pairwise comparisons as there were large differences in sample size for each factor (Stoline & Ury, 1979). Average response times to characteristics that loaded onto Unfriendly-Friendly were significantly faster than average response times to characteristics that loaded onto the Feeble-Formidable (p = .005), Unplanned-Planned (p = .005) and Adduction-Abduction (p = .022) factors. All other pairwise comparisons were non-significant (p > .05).

## Discussion

The results of the exploratory factor analysis indicated that a four-dimensional model with oblique rotation was the most appropriate model of the perception of dynamic human actions. These four factors were Feeble-Formidable, Unfriendly-Friendly, Unplanned-Planned and Adduction-Abduction and they represent the fundamental dimensions that form the conceptual space underlying action perception. The first two factors of Feeble-Formidable and Unfriendly-Friendly explained a larger proportion of the variance (22.1% and 22%, respectively) and the second two factors a smaller proportion (Unplanned-Planned 7.2% and Adduction-Abduction 8.6%). In addition, we observed broad groupings in the speed of the responses for each characteristic by the factors the characteristics substantially loaded onto. Ratings of the characteristics that loaded onto the Unfriendly-Friendly factor had significantly faster average response times than ratings of characteristics that loaded onto the other three factors, whilst there were no significant differences between comparisons of ratings loading onto the other factors. Although non-significant in this analysis, a general observation of the average response time for the different characteristics was that characteristics that loaded onto Feeble-Formidable were next fastest, followed by characteristics that loaded onto Unplanned-Planned, and that there was no distinctive pattern of average response time for characteristics that loaded onto Adduction-Abduction.

The two first factors (Feeble-Formidable and Unfriendly-Friendly) refer to more abstract action properties (Ciaramidaro et al., 2007; Van Overwalle & Baetens, 2009). These two fundamental action space dimensions have some parallels to those observed for face trait space (Oosterhof & Todorov, 2008; Sutherland et al., 2013), where dominance and trustworthiness are fundamental to face trait evaluation, and to the fundamental dimensions observed in a two-dimensional space of emotions (Bliss-Moreau et al., 2020; Feldman Barrett, 2017), namely arousal and valance. Our action space dimension of Feeble-Formidable represents similar characteristics for actions to what dominance does for face trait perception and arousal for emotion perception (indeed the action characteristic dominance loaded onto our Feeble-Formidable factor). Equally our action space dimension of Unfriendly-Friendly represents similar characteristics for actions to what trustworthiness does for face trait perception and valence does for emotion perception. In our study, the action characteristics of trustworthiness, approaching, and happiness loaded onto our Unfriendly-Friendly factor, whilst face trustworthiness, approachability and smiling loaded onto Sutherland et al. (2013) valence/trustworthiness factor. Although conceptual spaces are typically domain dependent, these similarities in the fundamental dimensions defining the conceptual spaces for these different domains may reflect some aspect of a single broader cross-domain conceptual space for the social evaluation of other individuals. While not directly equivalent, cross-modal conceptual spaces have already been observed for the perception of objects in both visual and tactile modalities (e.g., Gaissert et al., 2010), suggesting that conceptual spaces might not be restricted to the particular physical characteristics of the sensory information.

Our first fundamental action space dimension of Formidableness appears to reflect the assessment of an actor’s ability to implement their intentions. Determining this information from actions requires assessments of both simple movement characteristics – like action speed, fluency and power – but also more abstract characteristics, including the dominance and confidence of the actor. These more abstract social evaluations include assessments of others within the context of a social hierarchy, judgments that have shaped the evolution of the human mind (Cummins, 2000) and brain (Zink et al., 2008). Our second fundamental action space dimension of Friendliness also encompasses an evaluation of abstract qualities related to the intentions of the actor. Here the dimension is perhaps simpler to interpret, with either ends of the continuum representing whether the actor represents someone an individual would want to interact with or not. Social cooperation has numerous evolutionary benefits (Dugatkin, 2002), is pervasive between humans (Stevens & Hauser, 2004), and develops early (Warneken & Tomasello, 2007). Together, these two primary action space dimensions we identify here represent relatively abstract qualities of human actions that are related to fundamental evaluations that we need make to operate successfully within our complex social environment.

The two more minor factors Unplanned-Planned and Adduction-Abduction explain a smaller proportion of the variance in our model of action perception and both share weak correlations with the first two factors. Nevertheless, both a Parallel Analysis and model comparisons using EFA to CFA conversions showed that these were distinct separate factors underlying action perception. Importantly, and in contrast to the two primary dimensions underlying action space, these factors appear to underlie judgments that would be specific within the action domain.

The Unplanned-Planned dimension may reflect evaluations during ongoing predictions we make about the intentions and outcomes of other people’s actions. Actions are the principal way in which individuals influence their environment (including observers of these actions); we are constantly making predictions about how others will act and updating these predictions when they are violated (Flanagan & Johansson, 2003; Friston et al., 2011). Successful action prediction is important to successful social interactions (Sebanz & Knoblich, 2009), and develop during infancy (Monroy et al., 2019). Action prediction involves several brain regions, including early visual processing areas (Maffei et al., 2015) as well as the extended Mirror System (Kilner et al., 2007). And activity within these cortical regions may underpin the evaluation of actions on the Unplanned-Planned dimension of action space we identify here.

As with the Unplanned-Planned dimension, the ‘Adduction-Abduction’ dimension appears to reflect evaluation of a quality that could largely be unique to the action domain. The characteristics assessed that load onto this dimension involve the movements of limbs or objects towards (ingesting, pulling, getting) and away (expelling, pushing, releasing) from the actor. These reflect movements that can only be executed by an animate agent. However, these types of body movements can covary with size changes in the image of the actor at the retina. Movement of limbs away from the body typically results in an increase in the area of the retina subtended by the actor, whilst limb movements towards the body will decrease this area. Similar size changes are also commensurate with movements towards and away from the observer, respectively, a property of all physical objects and not just actors. It remains to be seen whether the Adduction-Abduction dimension we observe here is entirely unique to the kinematics of dynamic human actions with respect only to the actor themselves, or also has some relationship with movements of the actor with respect to the observer. In the latter case, this dimension may also have some importance in the representation of the social relevance of the action to the observer. For example, approaching actors may afford potential social opportunities or represent threats, retreating actors will be less relevant to the observer.

The dimensions we have identified here show some relationships with the principal components that best explained semantic categorisation of actions in the Tucciarelli et al. (2019) behavioural study, although they cannot be directly mapped onto each other. The semantic categories that varied along their first principal component varied according to the type of change induced by the execution of their actions. With change of location at one extreme through change of internal state in the middle to change of external state at the other extreme. This component is not directly comparable to our Adduction-Abduction dimension; however, Adduction-Abduction does represent the way actors change the external state of their environment (through moving it towards or away from them). Tucciarelli et al.’s third component appeared to represent how an action might be directed towards another individual or not. There are some parallels here with our Unfriendly-Friendly dimension, where ‘friendly’ actions involve positive interactions with others, whilst actions executed alone are in the middle of this dimension. Unfriendly actions directed towards another individual lie at the other end of our dimension. Such unfriendly actions were not categorised in Tucciarelli et al.’s experiment, and so we don’t know where they may lie within their model. However, they may lie the other side of the actions executed alone to the ‘friendly’ actions that they tested; thus, their third component might best explain categorisation of actions along a friendly-alone-unfriendly continuum akin to our Unfriendly-Friendly dimension.

The differences between our four-dimensional model of action space and other attempts at evaluating action space, in particular Tucciarelli et al.'s (2019) three-dimensional model of action categorisation, can be explained by substantive differences in the methodologies used. Tucciarelli et al. (2019) required participants to arrange static images of actions according to similarity of ‘meaning’; this precluded action comparisons on other qualities, like the nature of the movements themselves, or perhaps more abstract qualities to do with actor intentions. In contrast, we asked participants to rate dynamic actions on 23 pre-determined characteristics that encompassed a greater range of ways that actions can be evaluated. Even greater are the differences between the way Thornton and Tamir's (2022) six-dimensional model of actions was determined and the way we measured action space here. Their participants evaluated action verbs, many of which could not be executed by a human actor. It thus remains to be determined to what extent their ACT-FAST model represents the perceptual qualities of dynamic human actions. An interesting comparison is with the work of Wurm and colleagues who have been investigating the organisation of the neural representations underlying the recognition of actions (e.g., Wurm et al., 2017, and reviewed in Wurm & Caramazza, 2022). We don’t think that the framework proposed by Wurm and Caramazza (2022) and the action space we identify in our study are mutually exclusive. These approaches address different aspects of a much broader idea of action understanding. Whist Wurm and Caramazza focus on the ‘recognition’ of different actions and the organisation of how this may be achieved within occipitotemporal cortex, we believe that our action space encompasses the derivation of other information from actions importantly the ‘way’ that actions are executed. Assessments of whether an action is executed for example in a powerful, purposeful or trustworthy fashion can be achieved from a large range of different recognisable actions that in themselves have different purposes. There will of course be a degree of interaction between these frameworks, and this will be particularly interesting to determine in the future. For example, for each of our 240 different actions we calculated their distance to the centre of our four-dimensional action space. Object-directed transitive actions were significantly (t_(238)_ = 3.81, p < .001) more likely to be located towards the centre of actions space than other actions. We might tentatively suggest that those object-based actions that lie to the centre (e.g., spinning an object, unfolding, moving an object, poking, rolling, laying etc.) might not provide information of particular adaptive value to the observer, whereas many actions that are person-directed are located in the periphery of action space (and are thus further apart) as they need to be discriminated quickly as they may provide information of much higher value.

The design of our study did not allow for particularly accurate measures of response times when evaluating different action characteristics, as it was not an explicit reaction-time task, tasks were conducted remotely on devices not configured for measuring reaction times, and participants were required to indicate their response only after the completion of each presented action. Nevertheless, we observed a clear grouping of response times for three of the factors, perhaps reflecting shared processing of the characteristics that loaded substantially onto each factor. Furthermore, average response times to characteristics that loaded onto the Unfriendly-Friendly factor were significantly faster than the average response times to the characteristics that loaded onto the other three factors. There are two possible explanations for this observation. First, these grouped response times reflect some aspect of the stimuli themselves. Possibly information on Unfriendliness (and Friendliness) may be available earlier following the onset of the stimulus resulting in the shortest response times for characteristics that loaded onto this factor. For example, body form information like actor posture will be available from the first stimulus frame and may allow early detection of the relevant characteristics, whilst information on the other factors may occur later as the actions unfold with time, and with the availability of motion information. Alternatively, reaction times to the different characteristics may reflect differences in their processing times. Their sequence may represent their relative importance to the observer: the degree of friendliness or unfriendliness of another individual is most critical, as this factor may cue the potential of a threat or not to the observer, and as such is detected quickest. The degree of formidableness and abduction of the action may then help us interpret how to respond to the other individual, whilst determining the degree of planning behind the action is potentially less important to the observer. The sequencing of the importance of the two principal dimensions of first friendliness and then formidableness can also be interpreted within an evolutionary framework (cf. Fiske et al., 2007). This proposal suggests that there are universal dimensions of social cognition of warmth and competence. Evidence from the face domain (e.g., Knutson, 1996; Oosterhof & Todorov, 2008; Zebrowitz & Montepare, 2008) has suggested that the evaluation of different cues from faces can provide important adaptive information to infer behavioural intentions and power hierarchies. However, the degree to which facial appearance provides accurate information of the underlying intentions and capabilities of individuals remains contentious (Todorov et al., 2015). In contrast, deriving adaptive information about whether an actor intends to cause harm and has the ability to do so from the dynamic actions of an individual may be more accurate than that from static faces (cf. Aviezer et al., 2012).

Although the model of action space found in the current experiment explains a relatively large proportion of the variance (59.9%), this experiment was dependent upon ratings of the 23 characteristics that had been predetermined. We believe that these characteristics were comprehensive, given they were driven by a larger independent subset of actions and action words. Nonetheless, the requirement of pre-determined characteristics means that the analysis and subsequent model were dependent upon the characteristics selected, which has the potential to produce a less representative model of action space. For example, the importance of Unplanned-Planned as a latent factor may be overstated due to the exploratory factor analysis method simply grouping the accidental-intentional and uncontrolled-controlled characteristics into a single latent factor, because they are generally much more like one another, compared to the similarities between the other 18 included characteristics. This may explain why Unplanned-Planned was extracted as the third factor despite explaining the smallest proportion of variance and having only two substantially loading characteristics, when the traditional threshold suggests that at least three substantial loadings are required for the factor to be considered robust (Costello & Osborne, 2005; Hogarty et al., 2005). Nonetheless, the intraclass correlation coefficient for this factor indicated that the inter-characteristic reliability was ‘good’, a Parallel Analysis (Patil et al., 2008, 2017) indicated that four factors should be extracted, and the EFA to CFA conversion analysis, and subsequent robustness checks, all found that a four-dimensional model was a better fit for the data than a two-dimensional model. Thus, although this factor has fewer substantially loading characteristics than traditional thresholds would permit, it appears to be an influential latent factor of action perception. Nonetheless, an alternative truly data driven method of determining action space, like multidimensional scaling (Ding, 2018), would help to confirm the factors we identify here.

Our measure of action space is based upon evaluations of actions conveyed by a grey, androgynous avatar without a face, real-world context, or objects or other people observable during transitive and social actions. This was to ensure participants focused solely on the nature of the action itself, without judging non-action qualities of the stimulus. The restriction of our stimuli to body and body movement information may have resulted in some errors in the recognition of a few actions, for example our ‘closing a box’ and ‘folding a piece of paper’ actions were not recognised by many (4% and 10%, respectively). We believe that this is likely to be due to the absence of the presence of a specific object for these apparently ‘pantomimed’ actions that may help during a recognition task (see OSM). However, the task in our study was for participants to rate the actions on a number of different characteristics, judgements that can be achieved without the presence of visible objects (e.g., Cutting & Kozlowski, 1977; Sverker Runeson & Frykholm, 1981; S. Runeson & Frykholm, 1983). The identification of our actions as specifically closing a box or folding a piece of paper is unlikely to have a substantive impact on how participants rate them as fast, threatening, angry, pro-social, etc. However, we examined how well actions were recognised on the appropriateness of the two-, three-, four- and five-factor models, by comparing models developed from data where actions were well recognised with those from data where actions were less easily recognised. Irrespective of the recognisability of the actions, the four-factor model was optimal, the factors remained the same, and there were few differences in the comparative appropriateness of the alternative models. Together this all points towards the four-factor model of action space appearing relatively invariant to the recognisability of our action stimuli.

We might intuitively recognise this phenomenon, for example where we observe someone and do not know what they are doing but can still tell whether they appear friendly or not. Crucially for this experiment, given that participants were only presented with the avatar performing the action rather than the label, the actions needed to be perceived as being humanistic, with the body performing the action having expected human dimensions, orientation and range of movement (Dittrich, 1993; Loucks & Pechey, 2016; Thurman & Lu, 2016), and this was achieved through the use of a psychometric puppet performing the motion captured actions.

It was impossible to perform motion capture of some unique actions, like swimming, skiing and driving a car. These typically sport-related actions can be found within databases where actions have been videoed but are not represented well within our set of 240 actions. It is currently unclear whether the inclusion of these more sports-related actions would result in the identification of additional fundamental action space dimensions. We might speculate that many of these actions would be identified as largely planned and formidable and often abducting, but potentially neither friendly nor unfriendly. In addition, actions that would be labelled the same can be performed in many ways, for example skiing anxiously, slowly and without fluency, compared to skiing confidently, fast and with fluency. As such, actions with the same label can vary to differing extents along the four dimensions identified in this study. The absence of some sport-related uncapturable actions only limits our action set if some versions of these actions contain movements that are perceived as substantially different from the range of movements of those actions that were included. However, many elements of excluded and included actions overlap, for example aspects of skiing overlap with walking, ducking and even dancing.

Action evaluation in the real world will involve the incorporation of many different sources of additional social, contextual, and other multimodal information. The action space we identify here is restricted to the domain of actions as defined by body posture and bodily movements of the large set of 240 actions that we recorded. The model we describe is an attempt to identify a context-independent dimensional model of the fundamental qualities underlying action representation. Although context is useful in and improves action identification (Marszalek et al., 2009), in the question of action perception we don’t necessarily require different models each optimised for a range of different contexts. In the event we had many context-dependent action spaces, they would likely have many overlapping dimensions, as indeed many contexts themselves overlap. A more parsimonious solution would be to have a single action space with a shape and dimensionality reflecting the statistics of the actions previously encountered. Whilst during different future social contexts, we might place different emphasis on discrimination along certain action qualities (cf. Hebart et al., 2018; Nastase et al., 2017). In threatening situations, we might require more focus on the dimensions of aggression and predictability, whereas friendly situations may require evaluation of more social factors. How we make sense of actions in the real world may depend upon the dynamic interplay between a context-independent conceptual space for actions as we have measured here and other relevant representational spaces, in order to understand the whole person. These would include face trait (Oosterhof & Todorov, 2008; Sutherland et al., 2013) and identity spaces (Valentine, 2005), spaces for objects and tools (Martin, 2007), and even auditory spaces (Gygi et al., 2007). The current experiment asked participants to explicitly evaluate actions. This procedure is relatively uncommon in real-world social environments, as such further experiments will be required to understand whether our implicit evaluations of other peoples’ actions are based upon an organisation of action space along the four dimensions we describe here.

Lastly, we conducted analyses that decimated the data by the action to characteristic ratio and number of participants used to calculate average ratings of action characteristics. This potentially provides useful information for future EFA analyses. These analyses suggest a minimum of 7:1 subjects to variables ratio (in this study the equivalent is the actions to characteristics ratio), and a minimum of seven participants is needed when using mean subject scores. These suggestions contribute to the previous literature that debates the best practice guidelines for EFA analyses. Importantly, they additionally show that the four-factor action space model we identify here remains optimal and robust to variations in the contributing data to the analyses.

In conclusion the current study found that action space could be best described by a 2 + 2 factor model of action perception. This consisted of two substantial factors of Formidableness and Friendliness, which parallel similar dimensions identified in both face trait space and emotion space, and perhaps contribute towards a more generalised social perception space. In addition, the two minor factors of Planned and Abduction, appear to be particularly action specific and could, respectively, represent the nature of predictions we make about other peoples’ actions and visual qualities of actions.

### Supplementary Information

ESM 1 (DOCX 591 kb)

## Data Availability

We report how we determined our sample sizes, all data exclusions (if any), all manipulations, and all measures in the study, and we follow JARS (Kazak, 2018). Data collected during this study, including the words for determining the action characteristics, the mean ratings of the actions against the 23 characteristics, the loadings of the actions against the four fundamental factors, and movies of the actions themselves are all available publicly at: https://osf.io/4vew8/. Data were analysed using R Studio (R Core Team, 2020), and the packages used are detailed in the data analysis section. This study’s design and its analyses were not pre-registered.
