# Supplementary material for: Four fundamental dimensions underlie the perception of human actions
Source: Atten Percept Psychophys. 2023 May 15;86(2):536–58. doi: 10.3758/s13414-023-02709-1 (PMC10185378; doi:10.3758/s13414-023-02709-1)
Supplement: Supplementary file 1 — (DOCX 591 kb) [file 13414_2023_2709_MOESM1_ESM.docx]

**Supplemental materials**

**List of action databases inspiring action selection**

Action selection was based upon an assessment of a diverse range of current action databases: Point-Light Action Corpus (Shipley & Brumberg, 2005); CAVIAR Test Case Scenario (Fisher, Santos-Victor, & Crowley, 2004); Inferring intentions from biological motion: A stimulus set of point-light communicative interactions (Manera, Schouten, Becchio, Bara, & Verfaillie, 2010); The 20BN-“something-something” Dataset V2 (Goyal et al., 2017); Depth-included Human Action video dataset (Lin, Hu, Cheng, Hsieh, & Chen, 2012); HMDB: a large human motion database for human motion recognition (Kuehne, Jhuang, Garrote, Poggio, & Serre, 2011); Learning Human Actions from Movies (Laptev, Marszalek, Schmid, & Rozenfeld, 2008); IAS-Lab Action Dataset (Munaro, Ballin, Michieletto, & Menegatti, 2013; Munaro, Michieletto, & Menegatti, 2013); The LIRIS human activities dataset (Wolf et al., 2014); NTU RGB+D 120 Action Recognition Dataset (Jun Liu et al., 2019; Shahroudy, Liu, Ng, & Wang, 2016); ShakeFive2 (Van Gemeren, Poppe, & Veltkamp, 2016); UCF101 - Action Recognition Data Set (Soomro, Zamir, & Shah, 2012). These action databases included a total of 619 different actions, and we reduced this number progressively to a final list of 240 actions. We did this through a multi-step process. First, we removed all actions that would be impossible to record, typically these were actions related to sports (e.g. swimming, driving a car, surfing). Second, from the remaining actions we removed all duplicated actions. This left us with just over 240 actions, eliminating the last few actions involved selecting one of two very similar actions (e.g. pushing large object and pushing medium object). We checked our final set of 240 actions to ensure that we had a heterogenous sample by counting the number of transitive and social actions (reported) and dominant body part (not reported as deciding this is somewhat subjective).

**Motion capture procedure and stimuli processing**

Actions were performed by four actors (2 females, aged 23 and 59; 2 males aged 25 and 58) from one family group (due to government restrictions in social interactions during the coronavirus pandemic). Following motion capture, all recordings were cleaned within the Perception Neuron proprietary software Axis Neuron (Perception Neuron, Noitom, Miami, Florida, USA) to ensure consistent model alignment, and to set joint stiffness, step stiffness, and step constraint to optimise sensor placement with respect to the floor and ensure a realistic looking action by eliminating out of place/impossible sensor recordings. This was important as to allow for the use of motion capture recordings presented on characters for gaming purposes (for example) the Axis Neuron software allows for motion capture recordings of actions to be more fluid, however for our purposes we required the actions to look as natural as possible. Anti-magnetism was also applied to the whole body to account for local magnetic fields. Finally, a smooth factoring, which countered issues with any minor shaking and shifting of sensors, was applied. The full action recordings were initially cropped from the first frame in which movement of the actor (distinct from the natural oscillation of the body) began until the final frame of the last meaningful action movement before the body returned to a neutral position. An additional 30 frames (500ms) were included after the end of the action as a buffer. Once adjusted the final 1440 actions were exported as BioVision (.bvh) files.

**Avatar development**

The avatar was designed in Unity 3D (Unity, San Francisco, CA. USA) based upon the standard format of a .bvh file with the hips as a root 3D shape and the other body parts as further connected 3D shapes. This allowed the action to be presented via the X, Y and Z rotation of each relative body part from the root of the hips (Meredith & Maddock, 2001). The avatar was initially designed similar to that used in Roether, Omlor, Christensen, and Giese (2009) so that the body appeared human-like, in 3 dimensions with a grey surface. However, no personal characteristics (e.g. age or gender) could be derived from the avatar (e.g. from overall body shape, surface texture, clothing, facial characteristics etc., see Figure S1).

To assess how participants perceived the gender of the avatar, we initially conducted a short study where participants (n=14) were showed a static image of the waving avatar and they were asked to write a short biography for the avatar. Participants responses to the short biography showed that the avatar was not consistently presumed to have any distinctive characteristics. However, 7 participants assumed the avatar was male, the other 7 made no assumptions about avatar sex, suggesting a bias in perceived sex. Waist-hip ratios are well known cues to body sex e.g. (Molarius, Seidell, Sans, Tuomilehto, & Kuulasmaa, 1999). We, therefore, conducted a second study where participants (n=14) altered the width of the components that make up the avatar’s torso (collar, chest, upper spine, middle spine, lower spine and hips) so that the avatar appeared to first appear stereotypically feminine, then masculine, then androgynous. The width of each torso component was averaged across participants to generate feminine, masculine and androgynous bodies (see Figure S1).

The feminine body had a waist-hip ratio of 0.795:1, the masculine 1.062:1, and the androgynous 0.941:1. To generate an avatar that was perceived as neither male nor female, the final avatar torso component widths were set to the average of the feminine and masculine bodies. Although the final avatar waist-hip ratio (0.918:1) was slightly more masculine than feminine compared to that obtained by (Molarius et al., 1999), it was slightly more feminine than the ratio obtained from the androgynous avatar. This was deemed to be a good balance between masculine and feminine body shapes, especially given that, perhaps more importantly. all our action stimuli would be in motion and dynamic cues to gender tend to dominate structural cues (Mather & Murdoch, 1994) when these cues are in conflict.


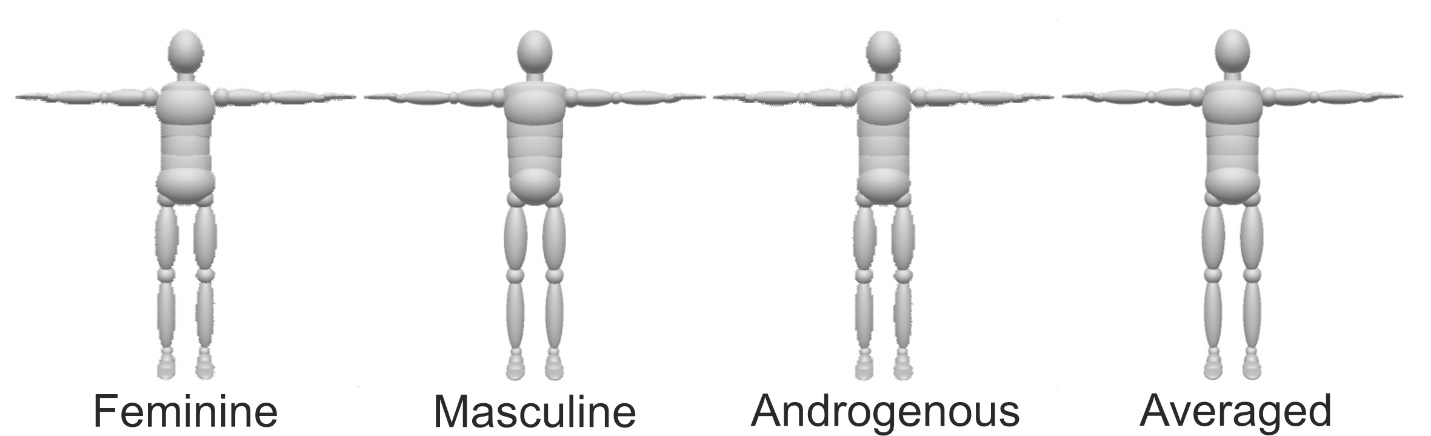


**Figure S1***. Images of the feminine, masculine, androgynous determined by participants, and final averaged avatar used during the rating experiment.*

**Action recognition**

Evaluation of participants’ ability to recognise the goals and intentions of the actors during the motion capture process was performed during an action naming task. Twelve example word-clouds (analysed in R Studio using the ‘wordcloud2’ package) based upon the names provided by participants for some actions distributed across the ‘recognisability spectrum’ are shown in Figure S2 to illustrate the diversity in action naming. Many actions were recognised by 100% of participants (see Figure S2 left column), these could be both non-transitive (dancing, celebrating) but also transitive actions (drinking, kicking a ball). For many of these easily recognisable actions, there was considerable agreement on names provided (e.g. dancing). In the middle of the spectrum (actions recognised by ~80%-87% of participants; see Figure S2 middle column) there were a range of different actions. Although these actions were recognised by the majority of people, some participants would either indicate a different goal directed action (e.g. putting something away instead of the correct: petting a dog), or only report descriptions of motor acts. With these later response types, we had no evidence that they had recognised the actor goals or intentions. For example, for the action where the actor was mopping or sweeping the floor, a few participants only described walking backwards, or twisting. At the lower end of the spectrum (see Figure S2 right column) were often transitive actions that involved interactions with objects. For example, hitting an object with a bat action was very poorly recognised, as the bat and object was not visible in the stimulus, participants instead typically recognised the action as throwing, an action involving very similar kinematics but different goals. Additions of objects, or other individuals, with which the actor was interacting would presumably help delineate the hitting action and some other poorly recognised transitive actions (e.g. attaching one object to another, 3% participants; poking an object so that it falls over, 7% participants; folding over a piece of paper 10%; taking objects from another person, 14% participants, etc.). Some other non-transitive actions were also poorly recognised. For example, the recognition of the laughing menacingly action appears particularly dependent upon movement of facial features that were not visible from our stimuli, instead participants reported small changes in head and body posture of the actor, but not the purpose of the action.


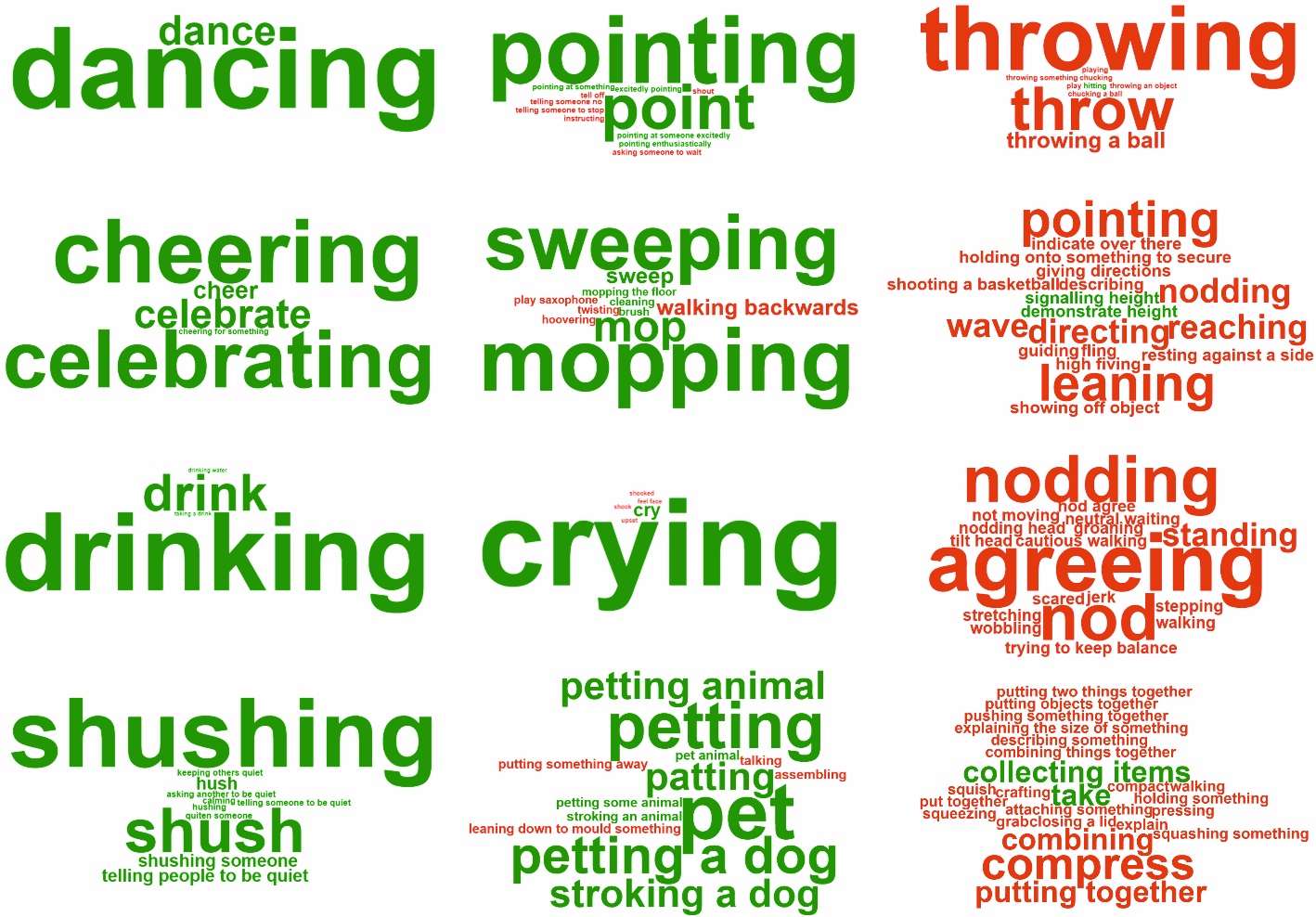


**Figure S2.** *Word-clouds of names allocated to example actions.* Size of names (or descriptions) are correlated with their frequency of allocation; names in green are matches to the respective action, whilst names in red are non-matches. Left column are word-clouds for example actions at the top of the recognisability spectrum [Dancing alone (100% participants); Celebrating and cheering while alone (100% participants); Drinking (100% participants]; Telling people to be quiet, shushing (100% participants)]. Middle column are word-clouds from example actions in the middle of recognisability spectrum [Pointing at something in an excited fashion (80% participants); Mopping or sweeping the floor (83% participants); Crying (83% participants); Crouching down and petting and animal (87% participants)]. Right column are word-clouds from example actions at the bottom of the recognisability spectrum [Hitting an object with a bat (3% participants); Indicating that an object is of a particular height (7% participants); Laughing menacingly (0% participants); Taking objects from another person (14% participants)].

**Selection of action characteristics**

List of action databases for selecting action characteristics: Actions as space time shapes (Gorelick, Blank, Shechtman, Irani, & Basri, 2007), the UCF Sports Action Data Set (Rodriguez, Ahmed, & Shah, 2008; Soomro & Zamir, 2014), the UCF YouTube Action Data Set (Jingen Liu, Luo, & Shah, 2009), the UCF101 Action Recognition Data Set (Kuehne et al., 2011), the Hollywood 2 human actions and scenes data set (Marszalek, Laptev, & Schmid, 2009), the KTH Recognising human actions dataset (Schuldt, Laptev, & Caputo, 2004), the UT interaction dataset (Ryoo & Aggarwal, 2010), and IXMAS Actions (Weinland, Özuysal, & Fua, 2010).

| **Table S3**  *Proportions of action words related to each characteristic. Characteristics are presented in the order determined during testing and are grouped by characteristic category* | | |
| --- | --- | --- |
| Characteristic | Characteristic category | Word proportion (%) |
| Avoiding/Approaching | Action goal | 5.00 |
| Releasing/Getting | Action goal | 4.20 |
| Hiding/Uncovering | Action goal | 3.00 |
| Ingesting/Expelling | Action goal | 1.60 |
| Breaking/Making | Action goal | 2.00 |
| Pulling/Pushing | Action goal | 5.70 |
| Lowering/Raising | Action goal | 6.10 |
| Removing/Adding | Action goal | 2.20 |
| Disapproving/Approving | Action intention | 0.90 |
| Ignoring/Communicating | Action intention | 3.10 |
| Rejecting/Desiring | Action intention | 3.70 |
| Accidental/Intentional | Action intention | 2.00 |
| Angry/Happy | Action intention | 4.30 |
| Anti-social/Pro-social | Action intention | 2.50 |
| Threatening/Protecting | Action intention | 2.10 |
| Straightening/Bending | Action movement | 2.80 |
| Uncontrolled/Controlled | Action movement | 6.90 |
| Hesitant/Fluent | Action movement | 2.70 |
| Low speed/High speed | Action movement | 3.20 |
| Weak/Powerful | Action movement | 5.20 |
| Anxious/Confident | Action trait | 2.10 |
| Subordinate/Dominant | Action trait | 1.20 |
| Untrustworthiness/Trustworthiness | Action trait | 0.40 |
| Object | Non-action category | 7.40 |
| Abstract concepts | Non-action category | 6.30 |
| Body part | Non-action category | 1.70 |
| Personality trait | Non-action category | 1.60 |
| Person descriptor (inc. nationality, occupation) | Non-action category | 3.00 |
| Location/Place | Non-action category | 1.20 |
| Sport | Non-action category | 7.80 |
| Unclassifiable | Non-action category | 0.80 |

**Additional demographic data**

**Table S4**

*Demographic information for each characteristic.*

| **Characteristic** | **Gender** | | | **Age** | |
| --- | --- | --- | --- | --- | --- |
|  | Female | Male | Prefer not to say | Mean | Standard deviation |
| Accidental - Intentional | 8 | 2 |  | 23 | 12.32 |
| Angry - Happy | 5 | 5 |  | 27.9 | 8.14 |
| Anti-Social - Pro-Social | 3 | 7 |  | 24.9 | 8.77 |
| Anxious - Confident | 6 | 4 |  | 25.3 | 6.07 |
| Avoiding - Approaching | 1 | 9 |  | 29.2 | 11.40 |
| Breaking - Making | 2 | 8 |  | 23.6 | 5.10 |
| Disapproving - Approving | 8 | 2 |  | 26.8 | 8.32 |
| Hesitant - Fluent | 1 | 9 |  | 25 | 7.33 |
| Hiding - Uncovering | 5 | 5 |  | 30.4 | 14.86 |
| Ignoring - Communicating | 2 | 8 |  | 31.4 | 14.73 |
| Ingesting - Expelling | 2 | 8 |  | 26.2 | 7.48 |
| Lowering - Raising | 9 | 1 |  | 27.8 | 19.30 |
| Low-Speed - High-Speed | 2 | 8 |  | 24.7 | 4.08 |
| Pulling - Pushing | 4 | 6 |  | 22.1 | 4.48 |
| Rejecting - Desiring | 5 | 5 |  | 27.9 | 10.31 |
| Releasing - Getting | 4 | 6 |  | 25.1 | 7.25 |
| Removing - Adding | 3 | 7 |  | 23.7 | 5.64 |
| Straightening - Bending | 4 | 6 |  | 23.5 | 5.66 |
| Subordinate - Dominant | 2 | 8 |  | 31.3 | 17.70 |
| Threatening - Protecting | 6 | 4 |  | 22.8 | 2.82 |
| Uncontrolled - Controlled | 5 | 5 |  | 25.2 | 8.09 |
| Untrustworthy - Trustworthy | 1 | 8 | 1 | 22.4 | 5.30 |
| Weak - Powerful | 5 | 5 |  | 26.6 | 4.38 |
| **Total** | **93** | **136** | **1** | **25.95** | **9.63** |

**References**

Fisher, R., Santos-Victor, J., & Crowley, J. (2004). CAVIAR test case scenarios. Retrieved from <https://homepages.inf.ed.ac.uk/rbf/CAVIARDATA1/>

Gorelick, L., Blank, M., Shechtman, E., Irani, M., & Basri, R. (2007). Actions as space-time shapes. *IEEE transactions on pattern analysis and machine intelligence, 29*(12), 2247-2253. doi:10.1109/TPAMI.2007.70711

Goyal, R., Ebrahimi Kahou, S., Michalski, V., Materzynska, J., Westphal, S., Kim, H., . . . Mueller-Freitag, M. (2017). *The" something something" video database for learning and evaluating visual common sense.* Paper presented at the Proceedings of the IEEE International Conference on Computer Vision.

Kuehne, H., Jhuang, H., Garrote, E., Poggio, T., & Serre, T. (2011). *HMDB: a large video database for human motion recognition.* Paper presented at the 2011 International Conference on Computer Vision.

Laptev, I., Marszalek, M., Schmid, C., & Rozenfeld, B. (2008, June). *Learning realistic human actions from movies.* Paper presented at the 2008 IEEE Conference on Computer Vision and Pattern Recognition

Lin, Y.-C., Hu, M.-C., Cheng, W.-H., Hsieh, Y.-H., & Chen, H.-M. (2012). *Human action recognition and retrieval using sole depth information.* Paper presented at the Proceedings of the 20th ACM international conference on Multimedia.

Liu, J., Luo, J., & Shah, M. (2009). *Recognizing realistic actions from videos “in the wild”.* Paper presented at the 2009 IEEE Conference on Computer Vision and Pattern Recognition.

Liu, J., Shahroudy, A., Perez, M., Wang, G., Duan, L.-Y., & Kot, A. C. (2019). Ntu rgb+ d 120: A large-scale benchmark for 3d human activity understanding. *IEEE transactions on pattern analysis and machine intelligence, 42*(10), 2684-2701.

Manera, V., Schouten, B., Becchio, C., Bara, B. G., & Verfaillie, K. (2010). Inferring intentions from biological motion: a stimulus set of point-light communicative interactions. *Behavior research methods, 42*(1), 168-178. doi:10.3758/BRM.42.1.168

Marszalek, M., Laptev, I., & Schmid, C. (2009). *Actions in context.* Paper presented at the 2009 IEEE Conference on Computer Vision and Pattern Recognition.

Mather, G., & Murdoch, L. (1994). Gender discrimination in biological motion displays based on dynamic cues. *Proceedings of the Royal Society of London. Series B: Biological Sciences, 258*(1353), 273-279. doi:10.1098/rspb.1994.0173

Meredith, M., & Maddock, S. (2001). Motion capture file formats explained. *Department of Computer Science, University of Sheffield, 211*, 241-244.

Molarius, A., Seidell, J., Sans, S., Tuomilehto, J., & Kuulasmaa, K. (1999). Waist and hip circumferences, and waist-hip ratio in 19 populations of the WHO MONICA Project. *International journal of obesity, 23*(2), 116-125.

Munaro, M., Ballin, G., Michieletto, S., & Menegatti, E. (2013). 3D flow estimation for human action recognition from colored point clouds. *Biologically Inspired Cognitive Architectures, 5*, 42-51. doi:10.1016/j.bica.2013.05.008

Munaro, M., Michieletto, S., & Menegatti, E. (2013). *An evaluation of 3d motion flow and 3d pose estimation for human action recognition.* Paper presented at the RSS Workshops: RGB-D: Advanced Reasoning with Depth Cameras.

Rodriguez, M. D., Ahmed, J., & Shah, M. (2008). *Action mach a spatio-temporal maximum average correlation height filter for action recognition.* Paper presented at the 2008 IEEE conference on computer vision and pattern recognition.

Roether, C. L., Omlor, L., Christensen, A., & Giese, M. A. (2009). Critical features for the perception of emotion from gait. *Journal of Vision, 9*(6), 15-15. doi:10.1167/9.6.15

Ryoo, M. S., & Aggarwal, J. (2010). *UT-interaction dataset, ICPR contest on semantic description of human activities (SDHA).* Paper presented at the IEEE International Conference on Pattern Recognition Workshops.

Schuldt, C., Laptev, I., & Caputo, B. (2004). *Recognizing human actions: a local SVM approach.* Paper presented at the Proceedings of the 17th International Conference on Pattern Recognition, 2004. ICPR 2004.

Shahroudy, A., Liu, J., Ng, T.-T., & Wang, G. (2016). *Ntu rgb+ d: A large scale dataset for 3d human activity analysis.* Paper presented at the Proceedings of the IEEE conference on computer vision and pattern recognition.

Shipley, T. F., & Brumberg, J. S. (2005). Markerless motion-capture for point-light displays. *Retrieved April, 4*, 2005.

Soomro, K., & Zamir, A. R. (2014). Action recognition in realistic sports videos. In *Computer vision in sports* (pp. 181-208): Springer.

Soomro, K., Zamir, A. R., & Shah, M. (2012). UCF101: A dataset of 101 human actions classes from videos in the wild. *arXiv preprint arXiv:1212.0402*.

Van Gemeren, C., Poppe, R., & Veltkamp, R. C. (2016). *Spatio-temporal detection of fine-grained dyadic human interactions.* Paper presented at the International Workshop on Human Behavior Understanding.

Weinland, D., Özuysal, M., & Fua, P. (2010). *Making action recognition robust to occlusions and viewpoint changes.* Paper presented at the European Conference on Computer Vision.

Wolf, C., Lombardi, E., Mille, J., Celiktutan, O., Jiu, M., Dogan, E., . . . Sankur, B. (2014). Evaluation of video activity localizations integrating quality and quantity measurements. *Computer Vision and Image Understanding, 127*, 14-30. doi:10.1016/j.cviu.2014.06.014
